# Automation in microinjection for zebrafish pericardial space with image-based motion control and batch agarose microplate

**DOI:** 10.1371/journal.pone.0333369

**Published:** 2025-10-09

**Authors:** Hyuk-Jin Lee, Hyun-Kyu Lee, Sang-Won Lee, Ye-Won Son, Jun-Nyeong Shin, Sohee Kim

**Affiliations:** 1 System R&D Team, Zefit Inc., Daegu, South Korea; 2 Neural Interfaces & MicroSystems Lab., Department of Robotics and Mechatronics Engineering, Daegu Gyeongbuk Institute of Science and Technology, Daegu, South Korea; Radboud University Medical Centre, NETHERLANDS, KINGDOM OF THE

## Abstract

Microinjection enables the precise delivery of substances into specific areas of small animals, such as zebrafish, whose xenograft models can be a promising platform for developing rapid and personalized cancer therapies. However, manual microinjection exhibits experimental variability and low reproducibility, as it relies on the expertise of researchers. To address these problems, automated microinjection systems have been developed in recent years. In this study, we propose a microrobotic system based on an image recognition AI model that extracts key feature points to define the pericardial space in zebrafish larvae at 2 days post-fertilization. Using the geometric relationships among feature points, the system optimizes the glass capillary insertion motion for precise microinjection. We also introduced a batch agarose microplate that prevents dehydration while stabilizing the larvae, which improved the survival rate compared to the conventional plate (log-rank test, *p* < 0.0001). The proposed automation system achieved success rates of 80.8% (*n* = 1129) for microinjection and a 92.1% (*n* = 1143) for survival. Moreover, we successfully injected colorectal cancer cell lines (HCT116 and SW620) into the pericardial space, resulting in an engraftment success rate of 96.2% (*n* = 610). Our system exhibits higher success rates and reproducibility compared to manual microinjection, allowing even inexperienced researchers to perform stable injections. These results demonstrate that our system effectively enhances the efficiency and reproducibility of experiments involving zebrafish-based cancer research and xenograft model generation.

## Introduction

Cancer is a major contributor to death globally, with incidence nearly doubling and mortality rates increasing over the past two decades [1]. Certain cancers with poor prognosis require rapid and personalized treatment due to difficulties in early diagnosis [2,3] or the recurrence of tumors that develop resistance to existing therapies and exhibit significant heterogeneity [4–6]. Xenograft models are an appropriate approach for developing treatment strategies based on individual patient characteristics [7,8]. While laboratory mice are widely used as an animal model, their application is associated with significant challenges, including experimental complexity, high costs, and variability, as obtaining results often takes several months [9,10]. Moreover, immune modulation is required to prevent immune rejection of human-derived tumors [10,11]. A substantial number of tumor cells are needed for successful engraftment, which poses challenges when patient-derived samples are limited [12]. Imaging tumor progression in mice typically requires invasive procedures, which makes real-time observation difficult [13]. To complement the limitations of mouse-based models, zebrafish have gained attention as a suitable alternative xenograft model for high-throughput screening, due to their distinct advantages: (1) ease of obtaining in large numbers [14], (2) rapid generation of experimental results within a few days, reducing overall costs [15,18], (3) undeveloped adaptive immune system during the early larval stage, eliminating the need for immunosuppressive treatment [16], (4) transparency of larvae, enabling real-time, non-invasive tumor observation in vivo [17], (5) requirement of only a few hundred cells for model generation [18]. However, zebrafish xenograft models are traditionally created by manual microinjection, which can easily be influenced by laboratory conditions, researcher expertise, and fatigue from repetitive tasks. Therefore, recent studies have focused on improving experimental efficiency and data precision by automating both the microinjection process and image analysis in embryos [19–22] and larvae [23–28]. In this study, we aim to address the following key technical challenges to achieve successful automation.

(1) Immobilization of larvae during microinjection should be ensured while maintaining viability. Some studies used negative pressure pipettes, which provided a stable immobilization [23,24], but the need for additional equipment to generate negative pressure and the requirement of one-to-one pairing with each larva limit their suitability for high-throughput screening. In contrast, agarose-based microplates with grooves provide reliability for handling multiple larvae at once, making them a preferred approach in most studies [25–27]. However, microplates require that a minimal amount of water be supplied to keep the larvae securely positioned against buoyancy, which can create a harsh environment for larval respiration. We designed a dish-shaped agarose microplate with a main reservoir in which each groove is connected via narrow channels. This structure enables both stable immobilization and continuous hydration of the larvae, providing a more suitable environment for automated microinjection.

(2) Image-based motion control algorithms are required to determine the optimal target site for each larva and to optimize the microinjection entry point. Commonly preferred injection sites in zebrafish include the pericardial space (PCS), perivitelline space (PVS), duct of Cuvier (DoC), yolk sac, and hindbrain ventricle. Among these, PCS is the preferred site due to its high larval survival rate during microinjection and its ability to provide stable tumor engraftment [29]. In this study, we developed an AI model that automatically detects the optimal injection site by analyzing the geometric relationships among three distinctive points near the PCS from lateral-view images of larvae acquired using a stereomicroscope. Based on this model, we implemented an automated system that ensures the injection needle tip mounted on a robotic arm accurately reaches the target point. To validate the performance of the system, we evaluated the injection needle positioning precision, injection speed, and the accuracy of AI-recommended injection sites. Furthermore, we tested our automated system using phenol red, trypan blue, FITC-dextran, and two colorectal cancer cell lines to evaluate the potential use of the platform in microinjection-based xenograft modeling in zebrafish.

## Materials and methods

**Fig 1:**
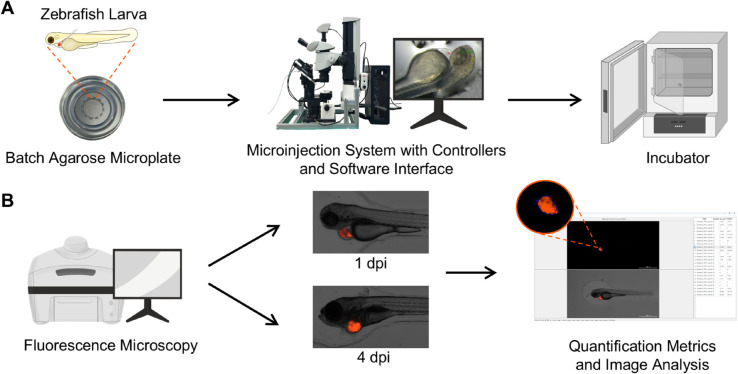
Experimental workflow overview. (A) Automated microinjection process at 2 dpf. (B) Fluorescence imaging and analysis at 1 and 4 dpi.

The overall experimental workflow consists of two main phases Fig 1: (A) automated microinjection, in which zebrafish larvae are positioned in a batch agarose microplate and injected with reagents or cells using an AI-guided robotic system; and (B) fluorescence imaging and computational analysis to visualize the injected cells and evaluate injection efficiency. Detailed incubation and imaging conditions are described in the following sections.

### Zebrafish preparation

#### Fish maintenance.

Wild-type (WT) AB strain zebrafish (*Danio rerio*) were used in this study. A total of 2,072 embryos were used in this study, including 160 embryos assigned to control groups. Embryos were obtained through natural spawning and subsequently maintained in E3 medium (5 mM NaCl, 0.17 mM KCl, 0.33 mM CaCl_2_, and 0.33 mM MgSO_4_, pH adjusted to 7.0–7.2) at 28.5 ^°^C under a 14 h light/10 h dark photoperiod [30,31].

At 1 days post-fertilization (dpf), embryos were allocated into experimental groups depending on the injection material and imaging requirements. All injection experiments were performed using embryos treated with 0.003% 1-phenyl-2-thiourea (PTU; P7629, Sigma-Aldrich) to inhibit pigmentation and ensure optical clarity. This approach was applied not only for cell injection experiments requiring fluorescence imaging but also for reagent injection experiments to maintain consistent experimental conditions.

Microinjections were performed at 2 dpf, when most embryos had hatched into larvae. Embryos that had not hatched spontaneously were manually dechorionated under a stereomicroscope using fine forceps.

Following microinjection, larvae injected with cells were transferred using a standard pipette tip into individual wells of a 24-well plate, each containing 1.8 mL of 0.1 mM PTU in E3 medium. This individual placement was performed to enable consistent tracking of each larva up to 4 days post-injection (dpi). The larvae were then incubated at 28 ^°^C for 1–2 hours to allow recovery from injection-induced stress and to maintain physiological stability. After this recovery period, the incubation temperature was increased to 34 ^°^C to promote the proliferation of human cancer cells and maintained until fluorescence imaging at 4 dpi.

#### Anesthesia protocol.

Tricaine methanesulfonate (MS-222; Sigma-Aldrich, E10521) was used to anesthetize larvae before microinjection at 2 dpf and imaging at 1 and 4 dpi. Larvae were immersed for ≥1 min in 400 μg/mL MS-222 until movement ceased. After microinjection (2 dpf) and the first imaging session (1 dpi), larvae were moved into a clean E3 dish and normal swimming resumed within 2–3 min.

#### Euthanasia and ethics approval.

Immediately after the final imaging session at 4 dpi, anaesthetised larvae were transferred to ice-cold E3 medium (0–2 ^°^C, no direct ice contact) and held for over 20 minutes. Death was confirmed by the absence of cardiac activity under a stereomicroscope. All procedures involving zebrafish were conducted in accordance with protocols approved by the Institutional Animal Care and Use Committee of Zefit Inc. (Approval No. ZEFIT-IACUC-25030501-0001).

### Injectable substances

#### Reagents.

The reagents used in this study were phenol red solution (P0290, Sigma-Aldrich), trypan blue solution (15250-061, Gibco), and fluorescein isothiocyanate-dextran (46944, Sigma-Aldrich).

#### Human cancer cell lines.

The human cancer cell lines used in this study were HCT116 (colorectal carcinoma) and SW620 (colorectal adenocarcinoma). Both were purchased from the Korean Cell Line Bank and cultured in RPMI 1640 medium (LM011-03, WELGENE) supplemented with 10% fetal bovine serum (FBS, Gibco), 1% penicillin-streptomycin (Gibco). The cells were maintained in a humidified atmosphere at 37 ^°^C and 5% CO_2_ and checked routinely for mycoplasma using a MycoAlert Mycoplasma Detection Kit (Lonza) according to the manufacturer’s instructions. Cells were harvested using 0.25% trypsin-EDTA (Gibco), stained with 4 *μ*M CM-DiI, and incubated on ice in the dark. Cells were then washed twice with DPBS (WELGENE), and the viability was assessed by trypan blue staining. Only samples with cell viability over 95% were used for microinjection. Following staining, labeled cells were centrifuged and resuspended in Geltrex (Thermo Fisher) serum-free medium to achieve the appropriate final concentrations for microinjection. The prepared cell suspensions were kept on ice until microinjection was performed to maintain cell viability and prevent premature aggregation.

### Automated injection system

#### Hardware.

The automated microinjection platform consists of four key hardware modules: (1) the batch agarose microplate designed to maintain larval viability and positioning during extended experimental procedures; (2) the microscope and imaging system for larval target detection and alignment verification; (3) the microinjection system for precise and reproducible injection; and (4) the computing and control unit that orchestrates real-time image analysis and hardware actuation. This modular design enables consistent microinjection performance across experiments, minimizes manual intervention, and improves throughput and reproducibility compared to conventional manual setups.

#### Batch microplate design.

Conventional agarose plates consist of only grooves without a reservoir and could not retain moisture effectively around the larvae. It does not provide sufficient time for researchers, especially novices, to complete the experiment. Manual rehydration is required for expanding the time but risks disrupting the larvae positioning during injection. Realignment is also a time-consuming process when the positioning is disrupted.

We developed the novel microplate to address these limitations. The microplate includes a central reservoir and the reservoir is connected to each groove. The design not only stabilizes larval positioning but also keeps each groove moist. We observed that the microplate with central reservoir maintained sufficient moisture to support larval viability over 1 hour.

**Fig 2:**
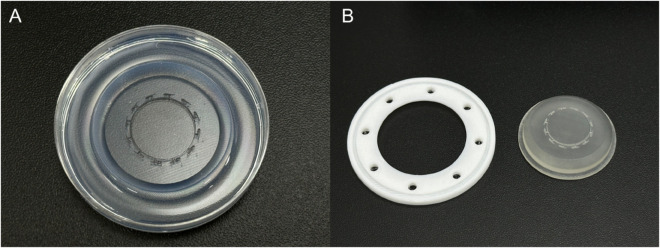
Design and fabrication of the batch microplate with a central reservoir. (A) Fabricated batch microplate featuring larva-sized grooves and a central reservoir. (B) 3D-printed supporting structure and resin mold used for shaping the batch microplate.

The batch microplate was fabricated by pouring a 2% agarose solution into the lid of a petri dish (10060, SPL) and molded using a custom-designed resin structure Fig 2. To prevent deformation during demolding, which can occur when the resin structure directly contacts the dish bottom while the agarose solidifies, a supporting structure made of polylactic acid was 3D-printed and placed beneath the resin structure to maintain a slight clearance from the dish bottom during the gelation process.

The resulting disk-shaped microplate contained 12 larva-sized grooves designed based on the mean body size of 2 dpf larvae [32], arranged circumferentially around the main reservoir. The reservoir was filled with E3 medium containing MS-222, which was gently distributed to each groove through microchannels to maintain sufficient hydration around each larva. Each 2 dpf larva was carefully placed into a single groove in a lateral orientation using a fine brush.

#### Microscope and imaging system.

The imaging system was integrated with a stereo microscope (M165, Leica) configured with a fixed 5× magnification. A 12-megapixel camera (Flexacam C1, Leica) was mounted on the microscope to capture images. An LED light source (KL300 LED, Leica) and a reflector were mounted for illumination.

A motorized rotation stage (8MR190-2-28, Standa) and a vertical adjustment stage (8MVT100-25-1, Standa) were incorporated into the system. The rotation stage was for sequential alignment of the larvae and the vertical stage enabled fine focus adjustments. Both ensured consistent image quality with sufficient field of view and focus.

#### Microinjection system.

A customized robotic arm (8-0347, customized, Standa) was used to control the position and angle of the injection needle. The needle was fabricated by pulling and cutting glass capillary tubes (TW100-4, World Precision Instruments). Following fabrication, the needle was securely held in place using a needle holder (Capillary Kit, World Precision Instruments), which was connected to a micropump (*μ*Pump, World Precision Instruments) for controlled fluid delivery.

#### Computing and control unit.

All key hardware components, including the needle actuator, rotation stage, and vertical adjustment stage, and the camera, were interfaced with an embedded computing system (Jetson AGX Orin, NVIDIA). The Jetson AGX Orin processes image data in real-time to compute the optimal injection point and transmits precise control signals to the microinjector, ensuring highly accurate and automated injections.

#### Software.

**Fig 3:**
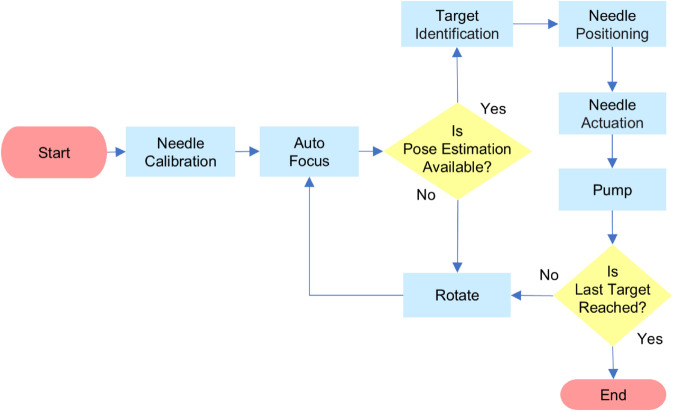
Overview of the automated microinjection workflow.

The microinjection workflow is shown in Fig 3. During needle calibration, the system adjusted the needle positioning accurately and verified fluid delivery via droplet formation. Upon successful calibration, the imaging system performed auto-focus to obtain a clear image for target identification.

An AI-based pose estimation model identified key points of the larva from the obtained image. Using these key points, the system computed the injection point as a weighted centroid within the PCS. The system then positioned the needle using XY-stage movement to align with a predefined, offset-adjusted target point.

After the needle positioning, the system drove the needle along a controlled trajectory at a preset angle to penetrate the target injection site. The system rotated to the next larval position and repeated the sequence until all larvae were processed.

#### Needle calibration.

Needle calibration was performed to align the imaging coordinate system$\left(p_{x},p_{y}\right)$ and the actuator coordinate system $\left(s_{x},s_{y}\right)$. The transformation factor *γ* was computed between the pixel changes $\left(\Delta p_{x},\Delta p_{y}\right)$ and the step changes $\left(\Delta s_{x},\Delta s_{y}\right)$, and was confirmed experimentally through multiple trials.

$$\gamma =\max(\gamma_{x},\gamma_{y}),\;\text{where }\gamma_{x}=\frac{\Delta s_{x}}{\Delta p_{x}},\;\gamma_{y}=\frac{\Delta s_{y}}{\Delta p_{y}}.$$ (1)

$$(\begin{array}{c} \Delta s_{x}\\ \Delta s_{y} \end{array})=\gamma \cdot (\begin{array}{c} \Delta p_{x}\\ \Delta p_{y} \end{array}).$$ (2)

#### AI-based target identification.

We selected a YOLO-based pose estimation model (YOLOv11m-Pose, Ultralytics) to detect the injection point and trained the model using 20,390 annotated images using a PC equipped with a GeForce RTX 3060 GPU (NVIDIA) [33]. These key points were manually labeled and then added to the training dataset to improve model accuracy.

**Fig 4:**
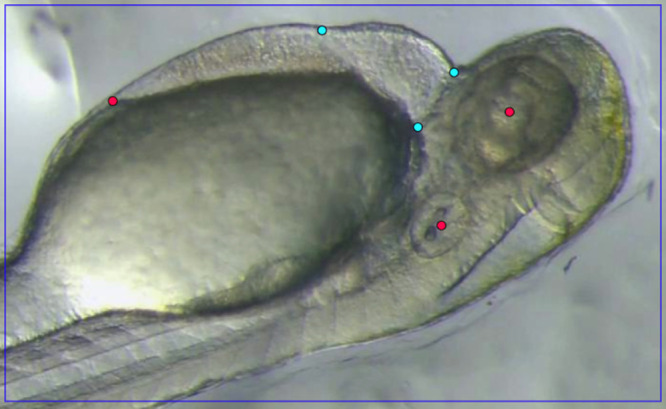
Annotation of three main points (cyan) and three auxiliary points (red).

An example of manually annotated training data used for model training is shown in Fig 4, illustrating main points corresponding to the three edges of the PCS and auxiliary points marking the centers of the eye and ear, and a specific edge of the PVS. The main points are directly used to compute the injection location, while the auxiliary points, which have strong features, help stabilize the training process.

**Fig 5:**
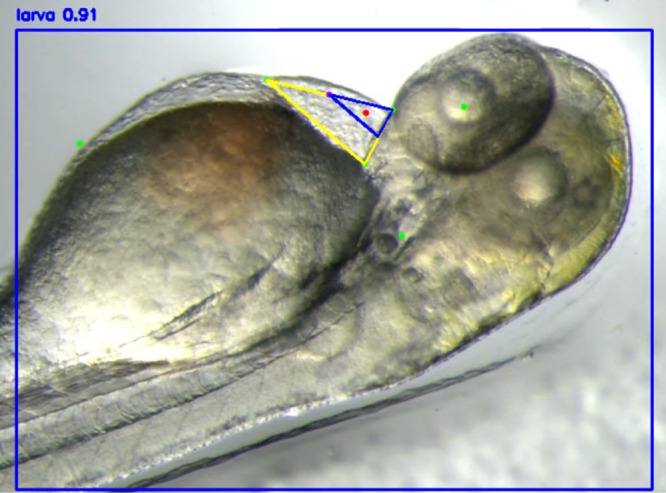
Weighted centroid (red) of the triangle formed by the main points. The main point nearest eye denotes $\left(x_{0},y_{0}\right)$.

In Fig 5, the PCS is defined as a yellow triangle formed by three typical points, $\left(x_{0},y_{0}\right)$, $\left(x_{1},y_{1}\right)$, and $\left(x_{2},y_{2}\right)$. To determine the optimal injection point $\left(x_{inj},y_{inj}\right)$, each of two vertices contributes half weight to its coordinates in the centroid calculation. This geometric adjustment was empirically derived to minimize displacement or rotation of the larva during the injection process. Accordingly, the final injection point is defined as the weighted centroid of the yellow triangle:

$$x_{inj}=\frac{1}{3}(x_{0}+\frac{\left(x_{0}+x_{1}\right)}{2}+\frac{\left(x_{0}+x_{2}\right)}{2}),$$ (3)

$$y_{inj}=\frac{1}{3}(y_{0}+\frac{\left(y_{0}+y_{1}\right)}{2}+\frac{\left(y_{0}+y_{2}\right)}{2}).$$ (4)

#### Injection execution.

**Fig 6:**
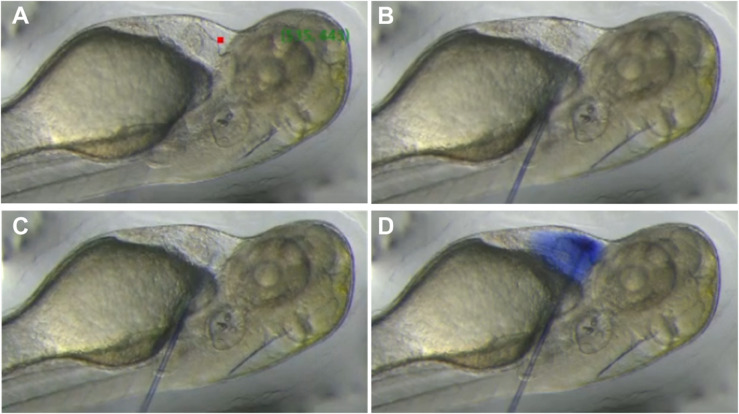
Step-by-step injection process performed by the automated microinjection system. (A) Target identification: the trained AI model identifies anatomical landmarks and selects the optimal injection site within the PCS. (B) Capillary moves to the offset-adjusted position in preparation for injection at the recommended site. (C) Capillary reaches the target location inside the PCS. (D) Reagent is successfully injected into the PCS, confirmed by visible trypan blue distribution.

The needle was mounted on the holder at the same angle used for manual injection, with its initial position denoted as $\left(x_{init},y_{init}\right)$. $\left(x_{off},y_{off}\right)$ is the distance from where the needle begins its descent to the injection point $\left(x_{inj},y_{inj}\right)$ within PCS and $\left(x_{adj},y_{adj}\right)$ represents the offset-adjusted coordinate where the needle must reach before its descent. The system moved the needle by (Δx,Δy) to the offset-adjusted coordinate. Once the needle reached the offset-adjusted coordinate, it descended at a predefined angle and punctured the skin. When its tip reached the injection point, the injection was carried out using a microinjection pump. The overall process is illustrated step-by-step in Fig 6.

$$(\begin{array}{c} x_{adj}\\ y_{adj} \end{array})=(\begin{array}{c} x_{inj}\\ y_{inj} \end{array})-(\begin{array}{c} x_{off}\\ y_{off} \end{array}),$$ (5)

$$(\begin{array}{c} \Delta x\\ \Delta y \end{array})=(\begin{array}{c} x_{adj}\\ y_{adj} \end{array})-(\begin{array}{c} x_{init}\\ y_{init} \end{array}).$$ (6)

### Fluorescence imaging and quantification

#### Fluorescence microscopy.

Fluorescence images were acquired using an automated microscope (BioTek Lionheart FX, Agilent). Agarose plates were prepared by pouring a 2% agarose solution into a petri dish, shaping it with a mold, and allowing it to solidify. A mold with a 4×10 array of larva-sized wells was designed and 3D-printed to ensure uniform placement. Each larva was then positioned laterally into a well and secured using methylcellulose, applied with a fine brush. Injected larvae were imaged at 1 dpi and 4 dpi.

#### Fluorescence quantification.

**Fig 7:**
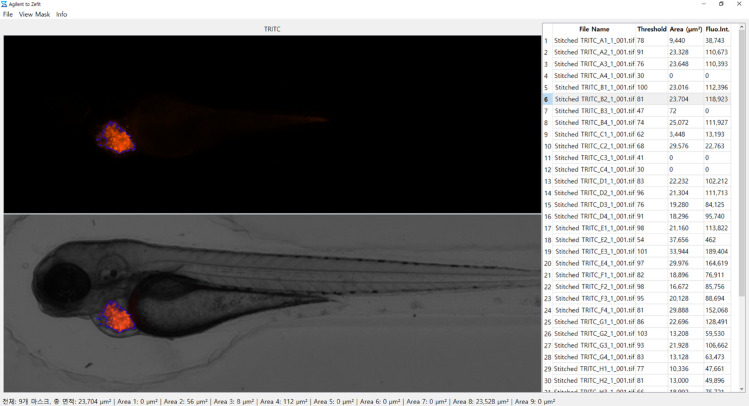
UI of the custom image analysis software showing contour detection and fluorescence quantification results.

To quantify fluorescence signals, we developed custom image analysis software using Python and OpenCV Fig 7. First, the software converts the RGB image into a grayscale image by applying a weighted sum of the red, green, and blue channels. Next, a manual threshold is applied using cv2.threshold to differentiate the fluorescence signal from background noise. Then, the contours of the fluorescence regions are detected using cv2.findContours, and the total fluorescence area is computed by summing the pixel areas enclosed within these contours with cv2.contourArea. Finally, the detected contours are visualized with cv2.drawContours, allowing real-time adjustment and verification of the results.

**Fig 8:**
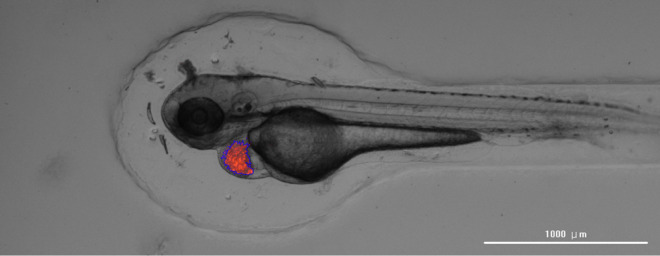
Detected fluorescence regions used for quantifying the total fluorescence area. Pixels exceeding the manually set intensity threshold are outlined in blue, representing the quantified fluorescence signals from injected cells.

The total fluorescence area *A*_*f*_ is determined by summing the number of pixels that exceed a manually set intensity threshold *T* Fig 8:

$$A_{f}=\sum_{i=1}^{N}1(I_{i}>T).$$ (7)

where *A*_*f*_ represents the fluorescence area in pixels, *N* is the total number of pixels in the image, *I*_*i*_ is the intensity value of the *i*-th pixel, *T* is the manually determined threshold, and 1(*I*_*i*_>*T*) is an indicator function that returns 1 if *I*_*i*_>*T* and 0 otherwise. This approach enables the precise quantification of fluorescence signals, facilitating the objective assessment of injection outcomes.

$$A_{\mu m^{2}}=A_{f}\cdot S_{\mu m^{2}/pixel}.$$ (8)

To convert the fluorescence area from pixels to actual physical units $\left(\mu m^{2}\right)$, the area conversion factor $S_{\mu m^{2}/pixel}$ was determined empirically using microscope calibration images with a known scale bar. Specifically, a pixel-to-micrometer conversion was performed by dividing the physical length represented by the scale bar (in micrometers) by its corresponding length in pixels. The squared value of this linear scaling was then used to compute the area conversion factor, enabling accurate computation of fluorescence area $A_{\mu m^{2}}$ in physical units.

We ensured consistent evaluation of fluorescence-based injection efficiency by standardizing image analysis and quantification, which allowed reliable comparison across different experimental conditions.

### Experimental design

#### Definition of quantitative metrics.

$$R_{sur1}=\frac{N_{sur1}}{N_{total}}\times 100\;(\%),$$ (9)

$$R_{sur4}=\frac{N_{sur4}}{N_{total}}\times 100\;(\%),$$ (10)

$$R_{suc}=\frac{N_{suc}}{N_{sur1}}\times 100\;(\%),$$ (11)

$$R_{eng}=\frac{N_{eng}}{N_{\left(suc\;\cap \;sur4\right)}}\times 100\;(\%).$$ (12)

In this study, we defined four quantitative metrics to evaluate the outcomes of injection experiments: larval survival rate until 1 dpi ($R_{sur1}$) and 4 dpi ($R_{sur4}$), injection success rate ($R_{suc}$), and cell engraftment rate ($R_{eng}$). The total number of injected larvae was denoted as $N_{total}$, the number of larvae with confirmed successful injection as $N_{suc}$, the number of larvae surviving until 1 dpi and 4 dpi as $N_{sur1}$ and $N_{sur4}$, and the number of larvae showing cell engraftment at 4 dpi as $N_{eng}$. Survival was assessed based on the number of larvae exhibiting normal morphology without decomposition or severe edema. Injection success was determined by confirming the presence of the injected material within the PCS. For reagent injection experiments, this was assessed immediately after microinjection either by direct visual inspection or, in the case of dextran reagents, by fluorescence microscopy. For cancer cell injections, injection success was assessed at 1 dpi by verifying the retention of cells within the PCS. All larvae were individually tracked and any larvae that died before the 1 dpi survival assessment were excluded from the count of successful injections when applicable. Cell engraftment was evaluated at 4 dpi based on whether the fluorescence area was maintained or increased compared to that observed at 1 dpi.

#### Evaluation of batch agarose microplate design on larval viability.

To assess the effect of batch agarose microplate design on larval survival, we compared two plate configurations: a conventional agarose microplate without a central reservoir (without reservoir group) and a batch agarose microplate containing a central reservoir (with reservoir group). Zebrafish larvae at 2 dpf were individually mounted on each plate, with 12 larvae assigned to each group, and subjected to time-lapse imaging using the automated injection system. Survival was monitored by recording the occurrence of cardiac arrest until all larvae in one group exhibited cardiac arrest.

#### Evaluation of automated system performance.

Prior to conducting the xenograft experiments, the performance of the automated microinjection system was evaluated across three domains: microinjection throughput, AI-based target identification accuracy, and microinjector control precision. Microinjection throughput and AI-based target identification accuracy were assessed concurrently. For this evaluation, two plates were prepared, each containing 12 larvae at 2 dpf. Each plate underwent five consecutive injection cycles, resulting in a total of 10 cycles and 120 injection attempts across 24 larvae. The throughput was calculated based on the time required for each cycle, and the target identification accuracy was evaluated by comparing the AI-predicted injection coordinates with manually annotated PCS. Microinjector control precision was assessed independently, without larvae, by measuring the positional error between user-defined target coordinates and the actual needle-tip positions using a calibrated 5-mm microgrid. Measurements were conducted at 24 distinct target locations within the working area.

#### Evaluation of injected reagents and cancer cells.

The effect of various injection substances on larval viability and injection success was assessed. Five substances—three visualization dyes (Phenol red, Trypan blue, FITC-dextran) and two human cancer cell lines (HCT116, SW620)—were tested to evaluate the generalizability of the injection process (Table 1).

**Table 1:** Sample allocation for each injection substance.

Substance/Control	Technical replicates	Biological replicates	*n*
Phenol red	3	3	108
Trypan blue	3	3	108
FITC-dextran	3	3	108
HCT116	3	3	108
SW620	3	3	108
Control (PTU)	-	3	60
**Total**			**600**

Each technical replicate consisted of 12 larvae per plate, and three plates were prepared for each biological replicate. The PTU control group was included to monitor baseline viability without injection and consisted of 20 larvae per biological replicate, without technical replicates.

#### Evaluation of novice and expert injection performance.

This experiment evaluated whether automation reduces the performance gap between novice and expert. To this end, novice and expert performed both manual and automated injections using the SW620 cell line under identical experimental conditions (Table 2).

**Table 2:** Sample allocation for novice and expert injection performance.

Person/Control	Technical replicates	Biological replicates	*n*
Expert (Auto)	4	3	120
Expert (Manual)	4	3	120
Novice (Auto)	4	3	120
Novice (Manual)	4	3	120
Control (PTU)	-	3	60
**Total**			**540**

Each technical replicate consisted of 12 larvae per plate, except for the fourth replicate, which consisted of 4 larvae. Four plates were prepared for each biological replicate. The PTU control group was included to monitor baseline viability without injection and consisted of 20 larvae per biological replicate, without technical replicates.

#### Evaluation of intra-novice and inter-novice injection performance.

This experiment evaluated whether automation reduces intra-novice and inter-novice variability among novices. To this end, three novice performed both manual and automated injections using the SW620 cell line (Table 3).

**Table 3:** Sample allocation for intra-novice and inter-novice performance.

Person/Control	Technical replicates	Biological replicates	*n*
Novice 1 (Manual)	4	4	160
Novice 1 (Auto)	4	4	160
Novice 2 (Manual)	4	4	160
Novice 2 (Auto)	4	4	160
Novice 3 (Manual)	4	4	160
Novice 3 (Auto)	4	4	160
Control (PTU)	-	4	80
**Total**			**1,040**

Each technical replicate consisted of 12 larvae per plate, except for the fourth replicate, which consisted of 4 larvae. Four plates were prepared for each biological replicate. The PTU control group was included to monitor baseline viability without injection and consisted of 20 larvae per biological replicate, without technical replicates.

#### Total number of larvae used.

Although the total sample count across all experimental conditions was 2,228 larvae, the actual number of larvae used was 2,072. This difference arose because the same experimental data obtained from individual larvae were reused in multiple evaluation analyses to reduce redundant animal use. Specifically, experimental data from 36 larvae for SW620 and 20 larvae for control in Table 1 were also incorporated into Table 2. Separately, data from 100 larvae in Table 2 (including control) were further included in Table 3.

#### Statistical analysis.

All statistical analyses were performed using GraphPad Prism (version 10.4.2 for Windows, GraphPad Software, Boston, Massachusetts USA) and R (version 4.0.5, The R Foundation for Statistical Computing, Vienna, Austria). Data are presented as mean ± standard deviation (SD), and the coefficient of variation (CV) was calculated as the percentage ratio of the SD to the mean within Prism. Both SD and CV calculations and visualizations were performed using Prism. Data normality was assessed using the Shapiro–Wilk test within Prism. Homogeneity of variances was assessed using Levene’s test within R. Since all data groups consisted of unpaired samples, Welch’s t-test was used when the assumption of normality was satisfied, and the Mann–Whitney U test was applied otherwise. A *p*-value of <0.05 was considered statistically significant, with significance indicated in the figures as *p < 0.05, **p < 0.01, ***p < 0.001, and ****p < 0.0001, with *ns* indicating not significant (p≥0.05).

## Results

### Evaluation of batch agarose microplate design on larval viability

**Fig 9:**
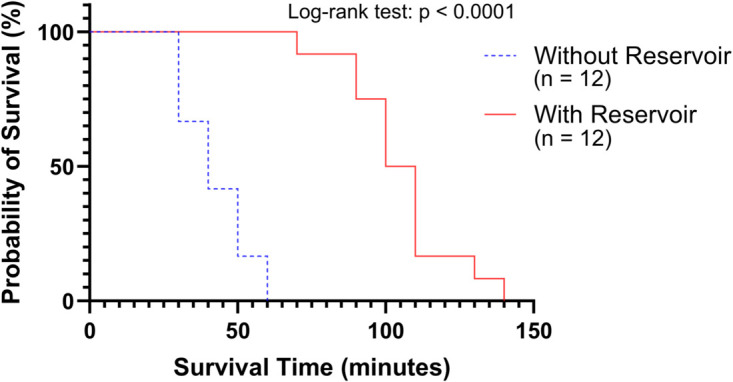
Larval survival time on different batch agarose microplate designs. Survival curves compare zebrafish larvae placed on agarose plates with and without a central reservoir under ambient conditions (20.4 ^°^C, 39% R.H.). All larvae on non-reservoir plates exhibited cardiac arrest within 60 minutes, whereas 100% survival was maintained up to 140 minutes in the reservoir group. The difference between groups was statistically significant (log-rank test, p < 0.0001, $\chi^{2}=23.669$). Each group included 12 larvae.

Under typical laboratory conditions, cardiac arrest began early in the without reservoir group (n = 12), with all larvae experiencing arrest within the first 60 minutes. In contrast, no cardiac arrest was observed in the with reservoir group (n = 12) during this period; the first case occurred at 70 minutes, and the last at 140 minutes. These results suggest that the central reservoir effectively delayed the onset of cardiac arrest and significantly improved larval viability throughout the injection procedure Fig 9. The data used to generate Fig 9 are available in the Supporting information (S1 Dataset).

### Evaluation of automated system performance

#### Microinjection throughput.

To evaluate the performance of the automated microinjection system prior to xenograft experiments, we measured the injection throughput. Two groups of 12 zebrafish larvae were prepared, and each group was subjected to five injection cycles (Cycle 1–5 and 6–10). In Cycle 9, two trials were excluded as outliers due to software-induced input delay and missing data. The average injection time per larva was 11.01 seconds (SD = 0.44 s), corresponding to an estimated throughput of approximately 327 larvae per hour. All data in Table 4 are available in the Supporting information (S2 Dataset).

**Table 4:** Average injection time and standard deviation per larval group.

Cycle	1	2	3	4	5	6	7	8	9	10
Mean (s)	10.92	11.18	11.16	10.97	10.97	10.99	10.93	10.82	10.93	11.24
SD (s)	0.30	0.64	0.37	0.24	0.17	0.13	0.12	0.45	0.49	0.77

SD: standard deviation; s: seconds. A total of 24 larvae were used.

#### AI-based target identification accuracy.

**Fig 10:**
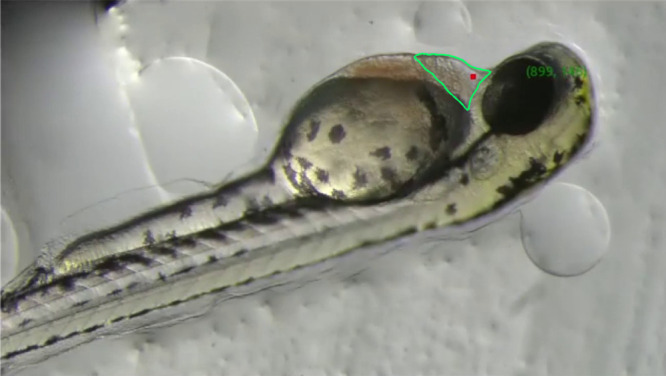
Visualization of AI-based target identification results. Red dots indicate the target injection positions predicted by the AI system on zebrafish larvae images. Manually annotated PCS regions are outlined for validation. The system identified injection sites within the PCS in all 24 independent trials.

The accuracy of AI-based target identification was assessed by verifying whether the injection points recommended by the image recognition software were located within the PCS. For this evaluation, the recommended injection sites for 24 independent zebrafish larvae were recorded, and the actual PCS region was manually annotated by a researcher for validation. In all 24 cases, the injection sites identified by the software were precisely located within the manually annotated PCS region, indicating that the trained AI model consistently achieved high accuracy in identifying the target injection site Fig 10.

#### Microinjector control precision.

The mean positional error was 18.46 μm (SD = 7.83 μm range: 0–34 μm), which is sufficiently small to ensure that the needle tip remains within the target region of the PCS during injection. While the actuator’s theoretical resolution is 1.25 μm, the observed accuracy remains consistent with this level when accounting for cumulative mechanical tolerances such as backlash, needle flexibility, and image-based measurement limitations. This high level of accuracy demonstrates that the robotic actuator can consistently and precisely position the needle at user-defined locations (Table 5). The data used to generate Table 5 are available in the Supporting information (S3 Dataset).

**Table 5:** Summary of positional error metrics.

Metric	Value
Number of measurements	24
Mean error	18.46
Standard deviation	7.83
Minimum error	0
Maximum error	34

All errors and deviations are expressed in micrometers (μm). A total of 24 larvae were used.

### Evaluation of injected reagents and cancer cells

**Fig 11:**
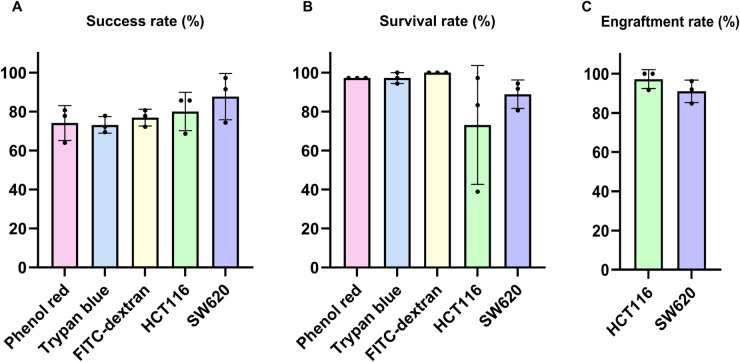
Injection outcomes for each substance using the automated method. (A) Injection success rates for each substance (B) Larval survival rates at 4 dpi. (C) Cell engraftment rates within the PCS at 4 dpi. Note that engraftment rates are only applicable to cell lines (HCT116 and SW620), not to reagent substances. Each dot represents the biological replicate. Bars represent the mean ± SD.

Fig 11 and Table 6 presents the results of individual biological replicates for each reagent and cell-injected group.

**Table 6:** Injection performance metrics for each substance and replicate condition.

	Replicate	Phenol red	Trypan blue	FITC-dextran	HCT116	SW620	Control group
$R_{suc}$ (%)	1	63.9 (23/36)	77.8 (28/36)	72.2 (26/36)	68.6 (24/35)	91.4 (32/35)	-
	2	80.6 (29/36)	69.4 (25/36)	80.6 (29/36)	85.7 (30/35)	97.2 (35/36)	-
	3	77.8 (28/36)	72.2 (26/36)	77.8 (28/36)	85.7 (30/35)	74.3 (26/35)	-
$R_{sur1}$ (%)	1	100 (36/36)	100 (36/36)	100 (36/36)	97.2 (35/36)	97.2 (35/36)	100 (20/20)
	2	100 (36/36)	100 (36/36)	100 (36/36)	97.2(35/36)	100 (36/36)	100 (20/20)
	3	100 (36/36)	100 (36/36)	100 (36/36)	97.2 (35/36)	97.2 (35/36)	100 (20/20)
$R_{sur4}$ (%)	1	97.2 (35/36)	97.2 (35/36)	100 (36/36)	83.3 (30/36)	80.6 (29/36)	100 (20/20)
	2	97.2 (35/36)	100 (36/36)	100 (36/36)	97.2 (35/36)	94.4 (34/36)	95.0 (19/20)
	3	97.2 (35/36)	94.4 (34/36)	100 (36/36)	38.9 (14/36)	91.7 (33/36)	95.0 (19/20)
$R_{eng}$ (%)	1	-	-	-	100 (20/20)	96.2 (25/26)	-
	2	-	-	-	100 (30/30)	84.8 (28/33)	-
	3	-	-	-	91.7 (11/12)	92.0 (23/25)	-

$R_{suc}$ : injection success rate; $R_{sur1}$ : survival rate at 1 dpi; $R_{sur4}$ : survival rate at 4 dpi; $R_{eng}$ : cell engraftment rate within the PCS. See Eq. (9)–(12) for the definitions of each metric. For each substance, each biological replicate consisted of 36 larvae, with four technical replicates performed per biological replicate. Table 6 provides the numerical data underlying the results shown in Fig 11.

#### Injection success rates.

Representative injection images and videos of each reagent and the cell-injected groups are provided in the Supporting Information. Phenol red is shown in S1 Movie, trypan blue in S2 Movie, FITC-dextran in S3 Fig and S3 Movie, HCT116 in S4 Movie, and SW620 in S5 Movie. Across all substances, the injection success rates ranged from 63.9%–97.2%. Most replicates exhibited a high injection success rate exceeding 70%. SW620 tended to show higher values than the others, but clear differences among substances were not observed.

#### Larval survival rates.

Representative images used to assess larval survival in reagent groups are provided in the Supporting Information. Phenol red is shown in S1 Fig, trypan blue in S2 Fig, and FITC-dextran in S4 Fig. The cell-injected groups were assessed for both survival and cell engraftment at 4 dpi. Across all substances, the survival rate at 1 dpi was uniformly high at 97.2%—100.0%. Also, the survival rate at 4 dpi was generally high 80.6%–100.0%. Only the third replicate of HCT116 showed notably low survival from 1 to 4 dpi as shown in Fig 11, which is presumed to result from anesthetic contamination during the transfer back to the well plate after imaging at 1 dpi.

#### Cell engraftment rates.

Engraftment rates were assessed only for the cell lines. Representative images of larvae engrafted with HCT116 are shown in S5 Fig and S6 Fig, and larvae engrafted with SW620 are shown in S7 Fig and S8 Fig. Their engraftment rates were consistently high, ranging from 84.8%–100.0%. The three reagent substances were not subject to engraftment assessment. All raw data shown in Table 6 are available in the Supporting information (S4 Dataset).

### Evaluation of novice and expert injection performance

To test the reproducibility and user-independence of the automated system, we compared the injection performance of a novice and an expert, using both manual and automated injection experiments. For each group, the average fluorescence area, standard deviation (SD), and coefficient of variation (CV) were calculated based on three independent replicates.

**Fig 12:**
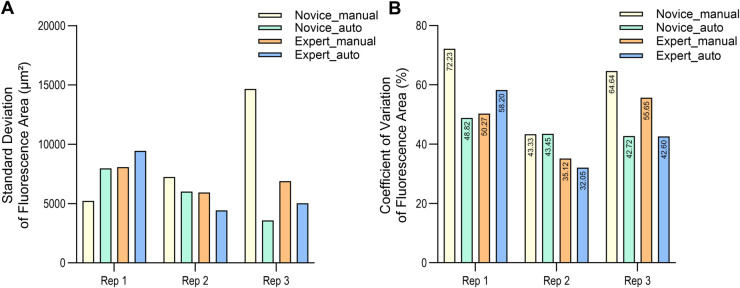
Fluorescence area variability in manual and automated microinjections by novice and expert. (A) Standard deviation of fluorescence area ($\mu m^{2}$) at 1 dpi across three biological replicates, comparing manual and automated injections by novice and expert. (B) Coefficient of variation (CV) of fluorescence area (%) at 1 dpi under the same conditions, illustrating relative variability normalized to mean fluorescence area across replicates. All experiments were conducted using the SW620. For each of the three biological replicates, four technical replicates were conducted, including three replicates with 12 larvae each and one replicate with 4 larvae, resulting in a total of 40 larvae per condition.

The results in Fig 12 and Table 7 demonstrated that, at 1 dpi in the second and third replicates, both the novice and expert achieved lower SDs in fluorescence area with automated injection compared to manual injection. In addition, except for the novice data in the second replicate, the CV was higher in manual injections than in automated injections across all conditions.

**Table 7:** Fluorescence area statistics at 1 dpi from manual and automated injections by novice and expert.

	Replicate	Novice		Expert	
		Manual	Auto	Manual	Auto
Mean ($\mu m^{2}$)	1	7,227	16,314	16,051	16,237
	2	16,704	13,849	16,886	13,814
	3	22,677	8,395	12,386	11,787
SD ($\mu m^{2}$)	1	5,221	7,965	8,070	9,449
	2	7,238	6,017	5,930	4,428
	3	14,658	3,586	6,893	5,021
CV (%)	1	72.23	48.82	50.27	58.20
	2	43.33	43.45	35.12	32.05
	3	64.64	42.72	55.65	42.60

For each of the three biological replicates, four technical replicates were conducted, including three replicates with 12 larvae each and one replicate with 4 larvae, resulting in a total of 40 larvae per condition. Table 7 provides the numerical data underlying the variability trends shown in Fig 12.

In the first replicate, this trend was not observed, as the SDs of both manual and automated injections were higher for the novice. Accordingly, when the relative distribution of the data was assessed using the CV in Table 7, automated injection had a 23.41% lower CV, which indicated superior data consistency.

**Fig 13:**
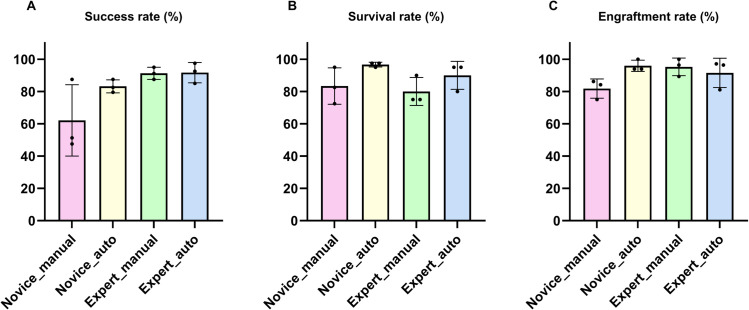
Injection outcomes in manual and automated methods performed by a novice and an expert (A) Injection success rates for manual and automated injections using the SW620 by a novice and an expert. (B) Larval survival rates at 4 dpi. (C) Cell engraftment rates within the PCS at 4 dpi. Each dot represents the biological replicate. Bars represent the mean ± SD.

In Fig 13 and Table 8, across all replicates, the manual injection success rate of the novice was equal to or lower than that of the expert. However, when using the automated system, the novice achieved success rates more comparable to manual injections of the expert.

**Table 8:** Performance metrics of injections performed by novice and expert.

		Novice		Expert	
	Replicate	Manual	Auto	Manual	Auto	Control group
$R_{suc}$ (%)	1	51.3 (20/39)	87.5 (35/40)	95.0 (38/40)	85.0 (34/40)	-
	2	87.5 (35/40)	82.5 (33/40)	87.5 (35/40)	97.5 (39/40)	-
	3	47.5 (19/40)	79.5 (31/39)	91.2 (31/34)	92.5 (37/40)	-
$R_{sur1}$ (%)	1	97.5 (39/40)	100 (40/40)	100 (40/40)	100 (40/40)	100 (20/20)
	2	100 (40/40)	100 (40/40)	100 (40/40)	100 (40/40)	100 (20/20)
	3	100 (40/40)	97.5 (39/40)	85.0 (34/40)	100 (40/40)	100 (20/20)
$R_{sur4}$ (%)	1	95.0 (38/40)	95.0 (38/40)	75.0 (30/40)	80.0 (32/40)	90.0 (18/20)
	2	82.5 (33/40)	97.5 (39/40)	90.0 (36/40)	95.0 (38/40)	100 (20/20)
	3	72.5 (29/40)	97.5 (39/40)	75.0 (30/40)	95.0 (38/40)	95.0 (19/20)
$R_{eng}$ (%)	1	84.2 (16/19)	93.9 (31/33)	96.4 (27/28)	96.4 (27/28)	-
	2	86.2 (25/29)	93.9 (31/33)	100 (31/31)	81.1 (30/37)	-
	3	75.0 (12/16)	100 (31/31)	89.3 (25/28)	97.1 (34/35)	-

$R_{suc}$ : injection success rate; $R_{sur1}$ : survival rate at 1 dpi; $R_{sur4}$ : survival rate at 4 dpi; $R_{eng}$ : cell engraftment rate within the PCS. Each biological replicate consisted of 40 larvae, with four technical replicates performed per replicate. Table 8 provides the numerical data underlying the results shown in Fig 13.

This trend is obvious when comparing the absolute number of successfully injected larvae, rather than relative injection success rate, as larvae that died between 0 and 1 dpi were excluded from the success rate calculation.

In experiments conducted by expert, automated injection showed a success rate similar to or higher than manual injection in all replicates except the first. Notably, in the third replicate, although the injection success rates were similar between the two methods, as described earlier, the absolute number of successfully injected larvae was greater for the automated injection.

$R_{sur1}$ were very high in most conditions, ranged from 85%–100%. The actual number of deceased larvae ranged from 0 to 6. At 4 dpi, both expert and novice achieved higher survival rates with automated injection compared to manual injection.

$R_{eng}$ in manual injections by novice ranged from 75%–86.2%, while the other conditions maintained higher rates between 81.1%–100%. Although these rates appear acceptable, the absolute number of engrafted larvae in novice manual injections was much lower due to reduced injection success and survival rates.

The corresponding data are depicted in Fig 13. All experimental data presented in Table 8 are available in the Supporting information (S5 Dataset).

### Evaluation of intra-novice and inter-novice injection performance

**Fig 14:**
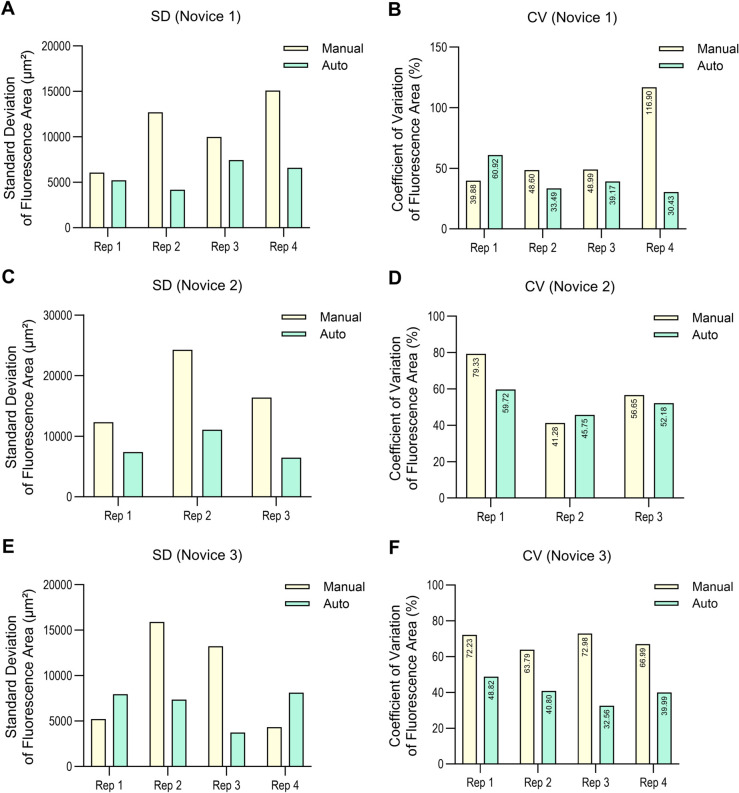
Fluorescence area variability after manual and automated microinjections by novices at 1 dpi. Standard deviation (SD) and coefficient of variation (CV) of fluorescence area in zebrafish larvae after manual and automated microinjections at 1 dpi are shown for three novice experimenters. (A) SD, novice 1. (B) CV, novice 1. (C) SD, novice 2. (D) CV, novice 2. (E) SD, novice 3. (F) CV, novice 3. All experiments were conducted using the SW620 cell line. For each of the three biological replicates, four technical replicates were conducted, including three replicates with 12 larvae each and one replicate with 4 larvae, resulting in a total of 40 larvae per condition.

As shown in Fig 14, the standard deviation for automated injection was generally lower than that for manual injection in most cases. However, in the first and fourth replicate of novice 3, the standard deviation was higher for automated injection. Although these results might suggest a lack of consistency in the automated injection data, an examination of the CV values in Table 9 revealed that the CV for automated injection was 23.41% lower in first replicate and 27% lower in fourth replicate. This indicates that manual injection in first and fourth replicate had lower reproducibility and data reliability, whereas automated injection provided greater consistency of injection and data reliability.

**Table 9:** Descriptive statistics of fluorescence areas after manual and automated microinjections by each novice.

	Replicate	Novice 1		Novice 2		Novice 3	
		Manual	Auto	Manual	Auto	Manual	Auto
Mean ($\mu m^{2}$)	1	15,219	8,591	15,507	12,398	7,227	16,314
	2	26,148	12,485	58,808	24,202	24,930	18,047
	3	20,395	19,006	28,931	12,380	18,131	11,480
	4	12,913	21,693	-	-	6,470	20,305
SD ($\mu m^{2}$)	1	6,069	5,233	12,301	7,404	5,221	7,965
	2	12,709	4,182	24,277	11,073	15,904	7,363
	3	9,991	7,446	16,390	6,460	13,231	3,737
	4	15,097	6,601	-	-	4,334	8,120
CV (%)	1	39.88	60.92	79.33	59.72	72.23	48.82
	2	48.60	33.49	41.28	45.75	63.79	40.80
	3	48.99	39.17	56.65	52.18	72.98	32.56
	4	116.9	30.43	-	-	66.99	39.99

For each of the three biological replicates, four technical replicates were conducted, including three replicates with 12 larvae each and one replicate with 4 larvae, resulting in a total of 40 larvae per condition. Table 9 provides the numerical data underlying the variability trends shown in Fig 14.

In Table 10, $R_{sur1}$ showed high survival rates between 87.5% and 100% across all novices and conditions. Additionally, when each novice was compared between manual and automated injection, survival rates were generally higher in the automated injection group. This trend was consistently observed in $R_{sur4}$ as well, and the difference was statistically significant with *p* = 0.0091 (Mann–Whitney U test; normality was confirmed for the automated group (*p* = 0.1615), but not for the manual group (*p* = 0.0221), based on the Shapiro–Wilk test; equal variance was confirmed by Levene’s test: *p* = 0.1283).

**Table 10:** Performance metrics of manual and automated injections by each novice.

	Replicate	Novice 1		Novice 2		Novice 3		
		Manual	Auto	Manual	Auto	Manual	Auto	Control Group
$R_{suc}$ (%)	1	87.5 (35/40)	77.5 (31/40)	40.5 (15/37)	74.4 (29/39)	51.3 (20/39)	87.5 (35/40)	-
	2	83.8 (31/37)	82.5 (33/40)	45.7 (16/35)	80.0 (32/40)	41.7 (15/36)	85.0 (34/40)	-
	3	75.0 (27/36)	87.2 (34/39)	56.4 (22/39)	81.1 (30/37)	60.0 (24/40)	94.9 (37/39)	-
	4	65.8 (35/38)	84.6 (33/39)	-	-	74.4 (29/39)	68.4 (26/38)	-
$R_{sur1}$ (%)	1	100 (40/40)	100 (40/40)	92.5 (37/40)	97.5 (39/40)	97.5 (39/40)	100 (40/40)	100 (20/20)
	2	92.5 (37/40)	100 (40/40)	87.5 (35/40)	100 (40/40)	90.0 (36/40)	100 (40/40)	100 (20/20)
	3	90.0 (36/40)	100 (40/40)	97.5 (39/40)	92.5 (37/40)	100 (40/40)	97.5 (39/40)	95.0 (19/20)
	4	95.0 (38/40)	97.5 (39/40)	-	-	97.5 (39/40)	95.0 (38/40)	100 (20/20)
$R_{sur4}$ (%)	1	92.5 (37/40)	97.5 (39/40)	85.0 (34/40)	95.0 (38/40)	95.0 (38/40)	95.0 (38/40)	90.0 (18/20)
	2	80.0 (32/40)	95.0 (38/40)	57.5 (23/40)	92.5 (37/40)	85.0 (34/40)	97.5 (39/40)	100 (20/20)
	3	75.0 (30/40)	89.7 (35/39)	92.5 (37/40)	85.0 (34/40)	85.0 (34/40)	92.5 (37/40)	95.0 (19/20)
	4	87.5 (35/40)	95.0 (38/40)	-	-	92.5 (37/40)	87.5 (35/40)	95.0 (19/20)
$R_{eng}$ (%)	1	97.1 (33/34)	93.3 (28/30)	84.6 (11/13)	100 (28/28)	84.2 (16/19)	93.9 (31/33)	-
	2	89.3 (25/28)	96.8 (30/31)	100 (7/7)	100 (30/30)	92.3 (12/13)	97.0 (32/33)	-
	3	77.3 (17/22)	100 (30/30)	100 (20/20)	96.3 (26/27)	85.7 (18/21)	100 (35/35)	-
	4	95.7 (22/23)	100 (33/33)	-	-	92.6 (25/27)	95.7 (22/23)	-

**Fig 15:**
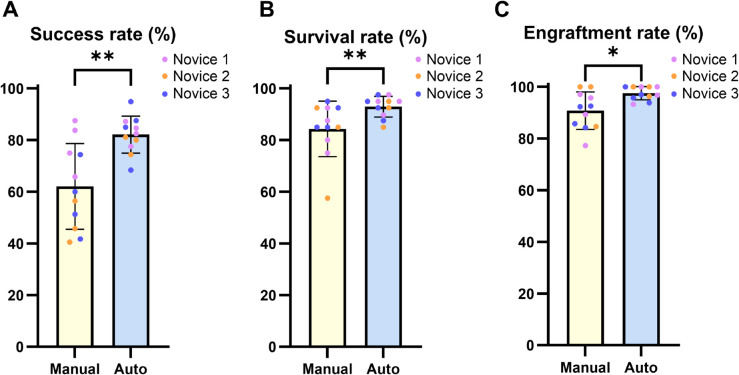
Injection outcomes in manual and automated methods performed by novices. (A) Injection success rates for manual and automated injections using the SW620 by three novices. (B) Larval survival rates at 4 dpi. (C) Cell engraftment rates within the PCS at 4 dpi. Each colored dot represents the biological replicate obtained by an individual novice. Bars represent the mean ± SD. Statistical significance was assessed using Welch’s t-test for (A). Mann–Whitney U test for (B) and (C). Significance is indicated as * *p* < 0.05 and ** *p* < 0.01.

$R_{suc}$ : injection success rate; $R_{sur1}$ : survival rate at 1 dpi; $R_{sur4}$ : survival rate at 4 dpi; $R_{eng}$ : cell engraftment rate within the PCS. For each of the four biological replicates, four technical replicates were conducted, including three replicates with 12 larvae each and one replicate with 4 larvae, resulting in a total of 40 larvae per condition. Table 10 provides the numerical data underlying the variability and performance comparisons by novice shown in Fig 15.

There was a marked difference in $R_{suc}$ between automated and manual injections for novices. The range of manual injection $R_{suc}$ among novices was 40.5%–87.5%, whereas automated injection improved this to 68.4%–94.9%. This improvement was especially pronounced in novice 2 and 3, and the difference was statistically significant with *p* = 0.025 (Welch’s *t*-test; normality was confirmed by Shapiro–Wilk test: *p* = 0.5199 for manual group and *p* = 0.9817 for automated group; unequal variance was confirmed by Levene’s test: *p* = 0.0087).

$R_{eng}$ generally achieved high levels in both manual and automated injections across all novices. However, when considering survival and success rates, which also affect engraftment, the absolute number of larvae with successful engraftment was higher with automated injection than with manual injection, and automated injection showed slightly better and more consistent engraftment rates as shown in Fig 15. This difference was statistically significant with *p* = 0.0184 (Mann–Whitney U test; normality was confirmed for the manual group (*p* = 0.6213), but not for the automated group (*p* = 0.0283), based on the Shapiro–Wilk test; unequal variance was confirmed by Levene’s test: *p* = 0.0129). All raw data presented in Table 10 are available in the Supporting information (S6 Dataset).

## Discussion

In this study, we developed an automated microinjection robot for creating xenograft models using zebrafish larvae. By combining pixel-to-micrometer conversion algorithms and motor control algorithms for the robot arm, we achieved precise guidance of the microinjection needle tip to a designated point on the image observed through a stereomicroscope. Also, we successfully automated the microinjection process by applying an enhanced larval embedding structure and an image processing system.

### Evaluation of batch agarose microplate design on larval viability

In Fig 9, we confirmed that the application of the batch agarose microplate significantly extended the survival time of larvae. The central reservoir in the plate clearly increased the amount of retained moisture and effectively prevented the larvae from drying out during the waiting or injection process, thereby reducing the duration of exposure to harsh environments and contributing to improved larval survival. In addition, the groove structure helped keep the larvae immobilized, which in turn enhanced the accuracy of target detection by the image-based motion control algorithms.

### Evaluation of injected reagents and cancer cells

We tested our automated system with three types of reagents, namely phenol red, trypan blue, and FITC-dextran, as well as two colorectal cancer cell lines, HCT116 and SW620. This experiment was conducted to overcome the limitations of previous studies that validated microinjection systems using only a single type of substance like phenol red. Our results clearly demonstrate that the performance of the automated system is not dependent on the type of substance being injected.

To compare all substances under identical environmental conditions, injections were performed using larvae that were born on the same day and from the same batch. Within a single biological replicate, three technical replicates were conducted for each substance, with a total of 36 larvae injected. The number of larvae used in each experiment was carefully determined based on the number of individuals that could be processed within a limited time frame. This consideration was made to minimize the potential variation in larval development, as zebrafish larvae at 2 dpf grow rapidly on an hourly basis, and even within the same biological replicate, growth differences may affect the outcomes.

To avoid potential bias from using a single batch of larvae that may favor specific outcomes, all substances were tested across three independent biological replicates, each conducted on different days. After injection, both reagent and cell-injected larvae were allowed to recover for 2 – 3 hours in a 28 ^°^C incubator before being transferred to a 34 ^°^C incubator for maintenance. The reason for using a temperature higher than conventional rearing conditions in this study is that human cancer cells such as HCT116 and SW620 require a temperature closer to human body temperature to proliferate stably. This selection was based on our preliminary experience showing that cell engraftment could not be reliably observed when larvae were maintained at 28 ^°^C. While 34 ^°^C is higher than standard rearing conditions and could be considered stressful for the larvae, the high survival rates observed in the control group suggest that it did not have a significant negative impact on larval viability.

We evaluated the injection success rate, larval survival between 0 – 1 dpi and 1 – 4 dpi, and cancer cell engraftment rate for each substance. In Table 6, the average injection success rates were 74.1% for phenol red, 73.1% for trypan blue, 75.0% for FITC-dextran, 80.0% for HCT116, and 87.7% for SW620. These results indicate that the injection process maintained high efficiency regardless of the type of substance used.

The average survival rate between 0 and 1 dpi was 100% for all reagents, 97.2% for HCT116, and 98.1% for SW620. These results confirm that the automated injection process did not cause fatal damage to the larvae.

The average survival rate between 1 and 4 dpi, which can be influenced by the toxicity of the injected substance and environmental conditions, showed a slight decrease compared to the survival rate between 0 and 1 dpi, but remained within acceptable experimental limits. An exceptionally low survival rate of 38.9% was observed in the third replicate of the HCT116. This was likely due to cross-contamination during the 1 dpi imaging step, possibly caused by residual anesthetic solution remaining in the pipette.

Finally, the engraftment rates were also high, with 98.4% observed for HCT116 and 90.5% for SW620. These results suggest that the combination of the automated injection system and appropriate experimental conditions enabled reliable and successful xenograft generation in zebrafish larvae.

### Evaluation of novice and expert injection performance

To evaluate how effectively the automated injection system can support novice users, we conducted a user-based comparison using cancer cells. We selected SW620, which had shown consistently favorable outcomes across all evaluation metrics. The comparison focused not only on injection success rate, larval survival rate, and cell engraftment rate, but also on the consistency of injections, as assessed by the standard deviation and coefficient of variation of the injected fluorescence area. The average injection volume was excluded from the comparison due to factors unrelated to user skill, such as slight differences in capillary diameter and variations in cell suspension viscosity with each capillary replacement. In addition, comparisons of statistics at 4 dpi were also excluded, as they could be influenced by individual larval condition and the state of the injected cells, rather than user proficiency.

In Fig 12 and Table 7, the novice user exhibited lower SD and CV in the second and third replicates when using the automated injection system. These results indicate that the automated injection system provided higher consistency and reproducibility for novice user. Although the SD was higher for automated injection in the first replicate, this was likely due to a difference in mean injection volume between the manual and automated groups. In this case, the lower CV observed in the automated group in first replicate still suggests higher precision and more consistent injection performance.

Furthermore, Table 8 shows that the novice achieved higher injection success rates, larval survival rates, and cell engraftment rates when using the automated system. These results demonstrate that the automated injection system developed in this study significantly improved the experimental accuracy and reproducibility for the novice.

In contrast, the expert showed similar or even higher SD and CV when using the automated injection system compared to manual injection. This suggests that the system may offer limited benefit to users who are already proficient in manual microinjection. However, this comparison was conducted with only three biological replicates, each consisting of 40 larvae, which may be insufficient to evaluate the effectiveness of the automated system for the expert. Considering the potential fatigue experienced by users during extended experiments [26], it is possible that the automated system could still offer advantages over manual injection by the expert, particularly in large-scale experiments. Nevertheless, further refinement of the automated system may be necessary to ensure that it provides meaningful benefits even for the expert.

### Evaluation of intra-novice and inter-novice injection performance

Finally, the comparison among novices was conducted to further validate the technical performance of the automated injection system. Each novice performed four biological replicates, although the fourth replicate of Novice 2 was excluded from the analysis due to an experimental error.

In Table 9 and Fig 14, the novices exhibited lower SD with automated injection compared to manual injection in most cases. Although Novice 3 showed lower SD with manual injection in the first and fourth replicates, this was likely due to absolute differences in injection volume between the manual and automated conditions. Even in these cases, the CV remained lower with automated injection, indicating superior overall injection precision. This suggests that the automated system helped novices maintain more consistent injection depth and positioning, thereby enabling more stable injections than with manual procedures.

Furthermore, as shown in Table 10 and Fig 15, statistically significant differences were observed between manual and automated injections among novices in terms of injection success rate, larval survival rate, and cell engraftment rate. These results clearly demonstrate that the automated system developed in this study provided improved injection performance when used by novices.

### Comparison with previous studies

In Table 11, we compare previous studies conducted on zebrafish embryos or larvae with the results of this study. Our automated system is the first to target the PCS as the injection site—a region preferred for xenograft model generation [29,34]. Through this study, we established the injection conditions and mechanisms required for successful microinjection automation within the zebrafish PCS, thereby laying the technical foundation for reliable system operation. Although PTU-treated larvae are widely used for their transparency in xenograft model development [35], their use in previous automated systems has not been reported. This study presents the first system capable of identifying the target region and performing successful microinjection even in PTU-treated larvae, thereby extending the applicability of automated microinjection systems.

**Table 11:** Performance comparison with existing automated microinjection systems.

Number of target	Time per target(s)	Success rate(%)	Survival rate(%)	Injection target	Target area	Injected materials	Larvae type	References
100	20.0	100	84.0	Embryos	Yolk	PCS2 vector	-	[20]
350	4.0	98.9	98.0	Embryos	Cytoplasm	Rhodamine B	-	[21]
50	12.0	96.0	100	Larvae	Heart ventricle, Atrium	Phenol-red	WT	[23]
160	24.6	95.0	98.7 (12 hpi)	Larvae	Yolk	Normal saline, Green fluorescent Microspheres	WT(AB strain)	[25]
88	13.9	92.1	93.8 (2 dpi)	Embryos, Larvae	Yolk	Unknown	WT	[26]
432	28.3	85.2	74.1	Larvae	Yolk	Fluorescent substances	WT	[27]
578	43.0	63.0	69.0 (3 dpi)	Larvae	DoC	Phenol-red, Trypan blue, Dextran, TILs, UM-UC-3, 2.5% Microsphere	WT(ABTL), Transparent casper, Tg(Fli: GFP) casper	[28]
375	47.0	45.0	95.0 (3 dpi)	Larvae	PVS	**MDA-MB-231, HCC1806**	WT(ABTL), Transparent casper, Tg(Fli: GFP) casper	[28]
113	36.5	57.5	90.5 (24 hpi)	Larvae	Hindbrain ventricle	Phenol-red, **Glioma cells**	WT(ABTL), Transparent casper, Tg(Fli: GFP) casper	[28]
1144	16.8	80.8	92.1 (4 dpi)	Larvae	PCS	Phenol-red, Trypan blue, Dextran, **HCT116, SW620**	WT(AB strain), PTU	This work

Human cancer cells used as injected materials are indicated in bold.

The injection speed of our system is 16.8 seconds per larva, which is the fastest reported among studies that have successfully performed cell injections. Injection time for all larvae used in this study is listed in the Supporting information (S7 Dataset). The system was tested using three reagents and two colorectal cancer cell lines, achieving an average injection success rate of 80.8% and an average survival rate of 92.1%, outperforming most previous studies. Also, our system was validated through a complete xenograft workflow, including cell engraftment, unlike many existing systems that focused solely on injection mechanics. Notably, larval survival was assessed at 4 dpi—an extended observation period compared to most previous studies—providing sufficient time to monitor tumor progression. These findings demonstrate not only the robustness and reliability of the automated injection process, but also its strong potential for application in preclinical cancer research.

Still, the current system needs to be expanded to support additional injection sites in order to become a broadly applicable automation platform for cancer research. In zebrafish xenograft models, various injection sites—such as the PVS, DoC, yolk, and hindbrain ventricle—are commonly preferred depending on the disease model, in addition to the PCS. In particular, the DoC is widely used in studies of cancer cell metastasis, and the hindbrain ventricle is highly applicable to brain cancer xenograft models [34,35]. Since each target site requires a different injection technique to minimize physical stress on the larvae and ensure stable cell delivery, it is necessary to establish site-specific microinjection protocols. As a follow-up study, we plan to develop an integrated microinjection automation system that can accommodate various target sites by replicating the techniques of experienced researchers, thereby expanding the scope of the current system beyond PCS as the sole injection site. Ultimately, this automated platform aims to fully replace manual xenograft procedures that rely on researcher skill, and to serve as a core infrastructure for high-throughput, large-scale zebrafish screening systems.

### Summary of findings

In summary, the experimental results of this study demonstrate that microinjection via an automated system consistently outperformed manual injection in key metrics such as injection success rate, larval survival rate, and cell engraftment success rate. These findings show that our system provides two adequate approaches: maintaining hydration through a main reservoir, and employing a weighted target selection method, which effectively minimizes larval damage by allowing the needle to avoid major blood vessels and reliably reach the PCS. In addition, our system significantly reduced the time required for microinjection and demonstrated lower experimental variability compared to manual injection. This performance advantage, especially evident when used by novice researchers, is considered a positive outcome as it minimizes user-dependent variability due to differences in skill level or fatigue, and ensures high reproducibility.

This study contributes to addressing the limitations of conventional microinjection processes, which require skilled researchers and a significant amount of time, and to proposing a method for leveraging the advantages of zebrafish as a model suitable for high-throughput screening. It also offers a promising path toward accelerating anticancer drug discovery through scalable, reproducible, and time-efficient xenograft studies.

## Supporting information

S1 Dataset Evaluation of batch agarose microplate design on larval viability. https://osf.io/q5v3c/files/osfstorage/6876012587882af364feb613
(XLSX)

S2 Dataset Evaluation of automated system performance: Microinjection throughput. https://osf.io/q5v3c/files/osfstorage/6876012650532c8c515a9b91
(XLSX)

S3 Dataset Evaluation of automated system performance: Microinjection control precision. https://osf.io/q5v3c/files/osfstorage/6876012937cdc0f727d32c82
(XLSX)

S4 Dataset Evaluation of injected reagents and cancer cells. https://osf.io/q5v3c/files/osfstorage/6895879fad00f6b3fb1b54e5
(XLSX)

S5 Dataset Evaluation of novice and expert injection performance. https://osf.io/q5v3c/files/osfstorage/68789a830e999809629c1f8e
(XLSX)

S6 Dataset Evaluation of intra-novice and inter-novice injection performance. https://osf.io/q5v3c/files/osfstorage/6895886c057ba611046b0c87
(XLSX)

S7 Dataset Comparison with previous studies. https://osf.io/q5v3c/files/osfstorage/6878a3c6b8976723cad32d3d
(XLSX)

S1 Fig Representative images of phenol red injected into the PCS at 4 dpi. Larvae injected with phenol red into the PCS using the automated microinjection system were imaged using the automated microscope (BioTek Lionheart FX, Agilent). All larvae shown in S1 Fig. were obtained from a single technical replicate, in which all 12 larvae were alive. Larval viability was determined based on the presence of a heartbeat and overall intact morphology; severe edema was considered indicative of death. At 4 dpi, phenol red had dispersed within the body and was no longer clearly visible, making it difficult to confirm successful injection. Therefore, the success of injection was confirmed using S1 Movie.
https://osf.io/q5v3c/files/osfstorage/68c9218ed79d3628ca41409b
(PDF)

S2 Fig Representative images of trypan blue injected into the PCS at 4 dpi. Larvae injected with trypan into the PCS using the automated microinjection system were imaged using the automated microscope (BioTek Lionheart FX, Agilent). All larvae shown in S2 Fig. were obtained from a single technical replicate, in which all 12 larvae were alive. Larval viability was determined based on the presence of a heartbeat and overall intact morphology; severe edema was considered indicative of death. At 4 dpi, trypan blue had dispersed within the body and was no longer clearly visible, making it difficult to confirm successful injection. Therefore, the success of injection was confirmed using S2 Movie.
https://osf.io/q5v3c/files/osfstorage/68c9218c8fb9447c641e482f
(PDF)

S3 Fig Representative fluorescence images of FITC-dextran injected into the PCS at 0 dpi. Larvae injected with FITC-dextran into the PCS using the automated microinjection system were imaged using the GFP filter of a digital microscope (CELENA^®^ S, Logos Biosystems Inc.). Imaging was performed immediately following microinjection, as the fluorescence intensity of FITC-dextran decreases within a few hours. All larvae shown in S3 Fig. were obtained from a single technical replicate, in which successful injection was observed in 11 out of 12 larvae. A video of the injection procedure is available in S3 Movie.
https://osf.io/q5v3c/files/osfstorage/68c92191cd6b208b5f3b813b
(PDF)

S4 Fig Representative images of FITC-dextran injected into the PCS at 4 dpi. Larvae injected with FITC-dextran into the PCS using the automated microinjection system were imaged using the automated microscope (BioTek Lionheart FX, Agilent). All larvae shown in S4 Fig. were obtained from a single technical replicate, in which all 12 larvae were alive. Larval viability was determined based on the presence of a heartbeat and overall intact morphology; severe edema was considered indicative of death.
https://osf.io/q5v3c/files/osfstorage/68ca0153204faa419f1e48a4
(PDF)

S5 Fig Representative fluorescence images of HCT116 injected into the PCS at 1 dpi. Larvae injected with HCT116 into the PCS using the automated microinjection system were imaged using the TRITC filter of an automated microscope (BioTek Lionheart FX, Agilent). All larvae shown in S5 Fig. were obtained from a single technical replicate, in which successful injection was observed in 9 out of 12 larvae. A video of the injection procedure is available in S4 Movie.
https://osf.io/q5v3c/files/osfstorage/68c92197fc82796659414143
(PDF)

S6 Fig Representative fluorescence images of HCT116 injected into the PCS at 4 dpi. Larvae injected with HCT116 into the PCS using the automated microinjection system were imaged using the TRITC filter of an automated microscope (BioTek Lionheart FX, Agilent). All larvae shown in S6 Fig. were obtained from a single technical replicate, in which all 12 larvae were alive. Engraftment was considered successful only when the fluorescent area at 4 dpi was maintained or increased relative to the area at 1 dpi. In this technical replicate, successful engraftment was observed in 9 out of 12 larvae.
https://osf.io/q5v3c/files/osfstorage/68c92197886836d6c61e481b
(PDF)

S7 Fig Representative fluorescence images of SW620 injected into the PCS at 1 dpi. Larvae injected with SW620 into the PCS using the automated microinjection system were imaged using the TRITC filter of an automated microscope (BioTek Lionheart FX, Agilent). All larvae shown in S7 Fig. were obtained from a single technical replicate, in which successful injection was observed in all 12 larvae. A video of the injection procedure is available in S5 Movie.
https://osf.io/q5v3c/files/osfstorage/68c9219c3f1362c8a553d88e
(PDF)

S8 Fig Representative fluorescence images of SW620 injected into the PCS at 4 dpi. Larvae injected with SW620 into the PCS using the automated microinjection system were imaged using the TRITC filter of an automated microscope (BioTek Lionheart FX, Agilent). All larvae shown in S8 Fig. were obtained from a single technical replicate, in which 11 out of 12 larvae were alive. Engraftment was considered successful only when the fluorescent area at 4 dpi was maintained or increased relative to the area at 1 dpi. In this technical replicate, successful engraftment was observed in 10 out of 11 larvae.
https://osf.io/q5v3c/files/osfstorage/68c9219c64ba17344e53d8aa
(PDF)

S1 Movie Representative movie of phenol red injection into the PCS. Movie documenting the automated microinjection of phenol red into the PCS. This movie was used to confirm successful injection, since phenol red was no longer clearly visible at 4 dpi in larvae shown in S1 Fig.
https://osf.io/q5v3c/files/osfstorage/6870689c5e162d4c47328083
(MP4)

S2 Movie Representative movie of trypan blue injection into the PCS. Movie documenting the automated microinjection of trypan blue into the PCS. This movie was used to confirm successful injection, since trypan blue was no longer clearly visible at 4 dpi in larvae shown in S2 Fig.
https://osf.io/q5v3c/files/osfstorage/6870689b6f768edcdb9168e5
(MP4)

S3 Movie Representative movie of FITC-dextran injection into the PCS. Movie documenting the automated microinjection of FITC-dextran into the PCS. This movie corresponds to the experiment shown in S3 Fig., where fluorescence imaging was performed immediately after injection due to the rapid decrease in FITC-dextran signal intensity.
https://osf.io/q5v3c/files/osfstorage/687068a1a086147a406bfece
(MP4)

S4 Movie Representative movie of HCT116 injection into the PCS. Movie documenting the automated microinjection of HCT116 cells into the PCS. This movie corresponds to the experiment shown in S5 Fig., in which successful injection was observed in 9 out of 12 larvae.
https://osf.io/q5v3c/files/osfstorage/687068a1996dbda4e370a340
(MP4)

S5 Movie Representative movie of SW620 injection into the PCS. Movie documenting the automated microinjection of SW620 cells into the PCS. This movie corresponds to the experiment shown in S7 Fig., in which successful injection was observed in all 12 larvae.
https://osf.io/q5v3c/files/osfstorage/68706a058bcf2e441991691b
(MP4)
