# Supplementary material for: Automation in microinjection for zebrafish pericardial space with image-based motion control and batch agarose microplate
Source: PLoS One. 2025 Oct 9;20(10):e0333369. doi: 10.1371/journal.pone.0333369 (PMC12510664; doi:10.1371/journal.pone.0333369)
Supplement: S2 Fig — Larvae injected with trypan into the PCS using the automated microinjection system were imaged using the automated microscope (BioTek Lionheart FX, Agilent). All larvae shown in S2 Fig. were obtained from a single technical replicate, in which all 12 larvae were alive. Larval viability was determined based on the presence of a heartbeat and overall intact morphology; severe edema was considered indicative of death. At 4 dpi, trypan blue had dispersed within the body and was no longer clearly visible, making it difficult to confirm successful injection. Therefore, the success of injection was confirmed using S2 Movie. https://osf.io/q5v3c/files/osfstorage/68c9218c8fb9447c641e482f (PDF) [file pone.0333369.s009.pdf]

S2 Fig.

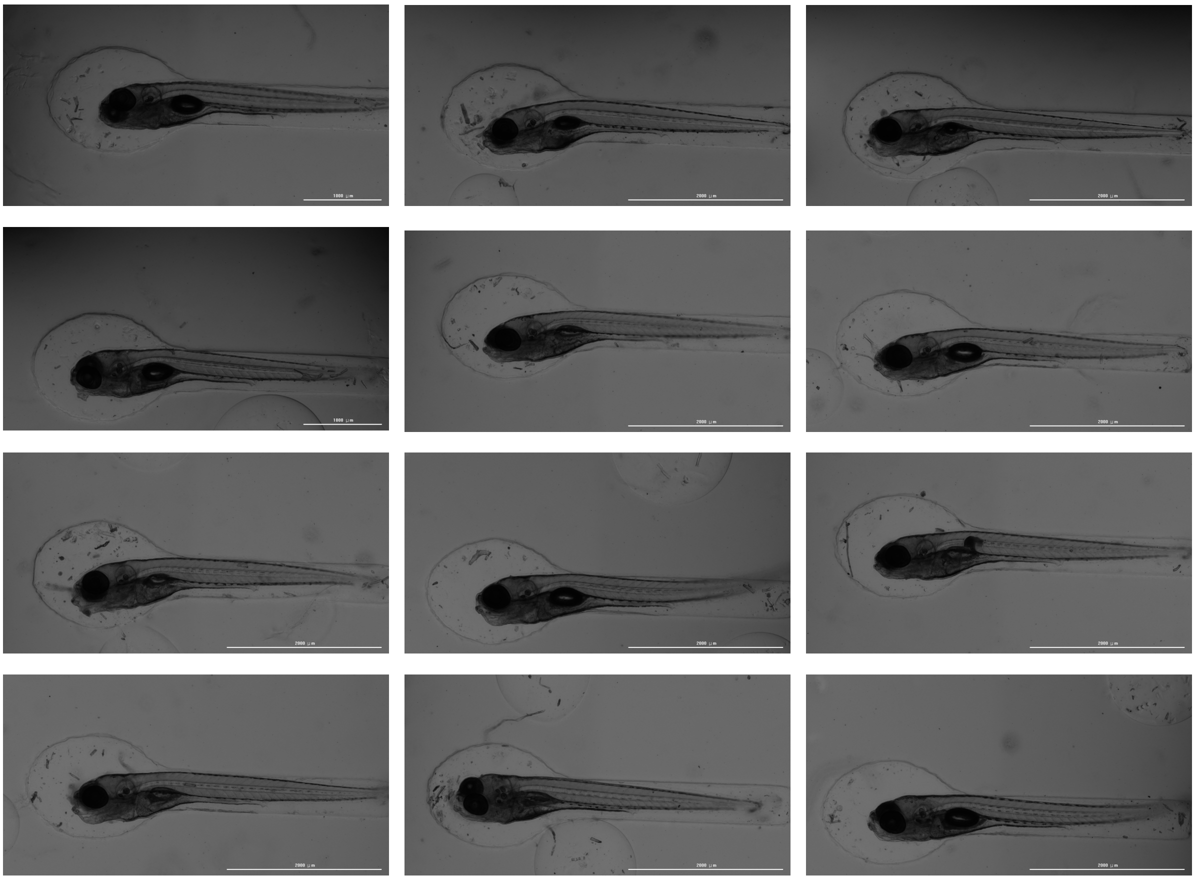

S2 Fig. Representative images of trypan blue injected into the PCS at 4 dpi. Larvae injected with trypan into the PCS using the automated microinjection system were imaged using the automated microscope (BioTek Lionheart FX, Agilent). All larvae shown in S2 Fig. were obtained from a single technical replicate, in which all 12 larvae were alive. Larval viability was determined based on the presence of a heartbeat and overall intact morphology; severe edema was considered indicative of death. At 4 dpi, trypan blue had dispersed within the body and was no longer clearly visible, making it difficult to confirm successful injection. Therefore, the success of injection was confirmed using S2 Movie.
