# Supplementary material for: Automation in microinjection for zebrafish pericardial space with image-based motion control and batch agarose microplate
Source: PLoS One. 2025 Oct 9;20(10):e0333369. doi: 10.1371/journal.pone.0333369 (PMC12510664; doi:10.1371/journal.pone.0333369)
Supplement: S3 Fig — Larvae injected with FITC-dextran into the PCS using the automated microinjection system were imaged using the GFP filter of a digital microscope (CELENA® S, Logos Biosystems Inc.). Imaging was performed immediately following microinjection, as the fluorescence intensity of FITC-dextran decreases within a few hours. All larvae shown in S3 Fig. were obtained from a single technical replicate, in which successful injection was observed in 11 out of 12 larvae. A video of the injection procedure is available in S3 Movie. https://osf.io/q5v3c/files/osfstorage/68c92191cd6b208b5f3b813b (PDF) [file pone.0333369.s010.pdf]

S3 Fig.

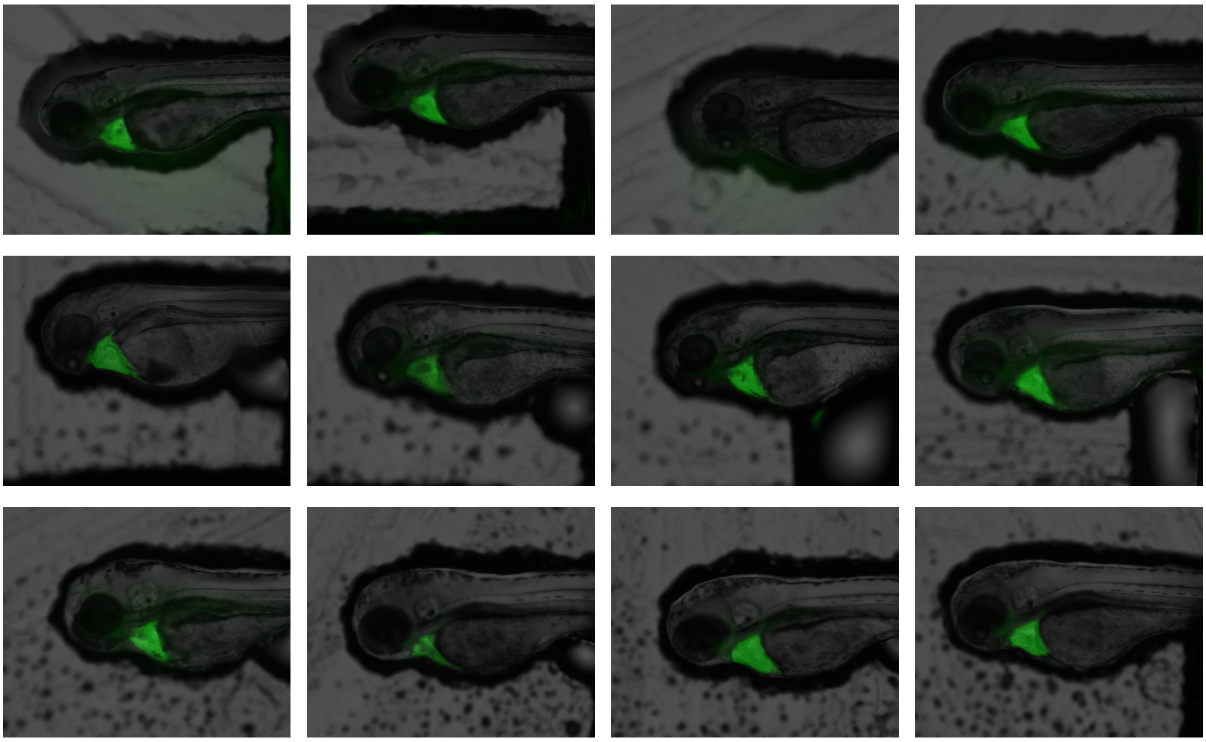

S3 Fig. Representative fluorescence images of FITC-dextran injected into the PCS at 0 dpi. Larvae injected with FITC-dextran into the PCS using the automated microinjection system were imaged using the GFP filter of a digital microscope (CELENA® S, Logos Biosystems Inc.). Imaging was performed immediately following microinjection, as the fluorescence intensity of FITC-dextran decreases within a few hours. All larvae shown in S3 Fig. were obtained from a single technical replicate, in which successful injection was observed in 11 out of 12 larvae. A video of the injection procedure is available in S3 Movie.
