# Supplementary material for: Automation in microinjection for zebrafish pericardial space with image-based motion control and batch agarose microplate
Source: PLoS One. 2025 Oct 9;20(10):e0333369. doi: 10.1371/journal.pone.0333369 (PMC12510664; doi:10.1371/journal.pone.0333369)
Supplement: S4 Fig — Larvae injected with FITC-dextran into the PCS using the automated microinjection system were imaged using the automated microscope (BioTek Lionheart FX, Agilent). All larvae shown in S4 Fig. were obtained from a single technical replicate, in which all 12 larvae were alive. Larval viability was determined based on the presence of a heartbeat and overall intact morphology; severe edema was considered indicative of death. https://osf.io/q5v3c/files/osfstorage/68ca0153204faa419f1e48a4 (PDF) [file pone.0333369.s011.pdf]

**S4 Fig.**

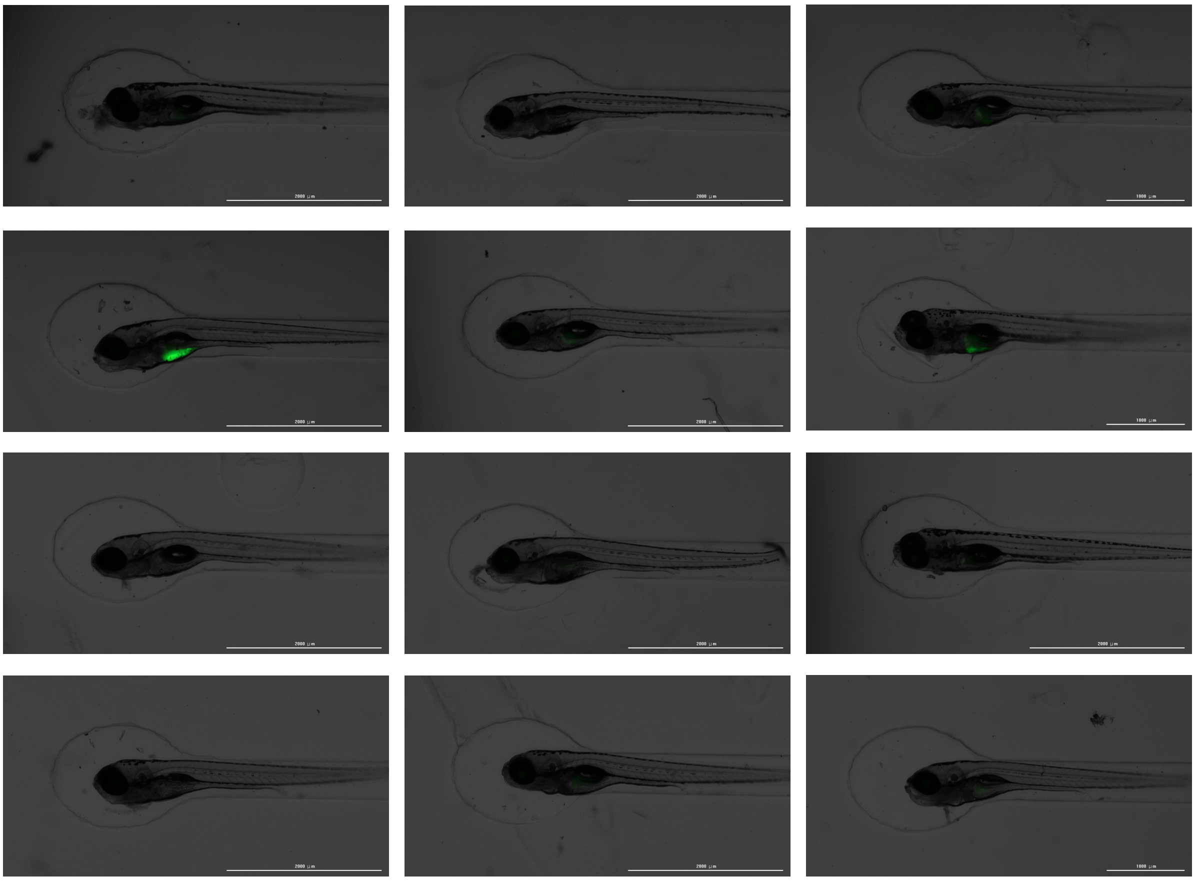

**S4 Fig. Representative images of FITC-dextran injected into the PCS at 4 dpi.** Larvae injected with FITC-dextran into the PCS using the automated microinjection system were imaged using the automated microscope (BioTek Lionheart FX, Agilent). All larvae shown in S4 Fig. were obtained from a single technical replicate, in which all 12 larvae were alive. Larval viability was determined based on the presence of a heartbeat and overall intact morphology; severe edema was considered indicative of death.
