# Supplementary material for: Automation in microinjection for zebrafish pericardial space with image-based motion control and batch agarose microplate
Source: PLoS One. 2025 Oct 9;20(10):e0333369. doi: 10.1371/journal.pone.0333369 (PMC12510664; doi:10.1371/journal.pone.0333369)
Supplement: S5 Fig — Larvae injected with HCT116 into the PCS using the automated microinjection system were imaged using the TRITC filter of an automated microscope (BioTek Lionheart FX, Agilent). All larvae shown in S5 Fig. were obtained from a single technical replicate, in which successful injection was observed in 9 out of 12 larvae. A video of the injection procedure is available in S4 Movie. https://osf.io/q5v3c/files/osfstorage/68c92197fc82796659414143 (PDF) [file pone.0333369.s012.pdf]

S5 Fig.

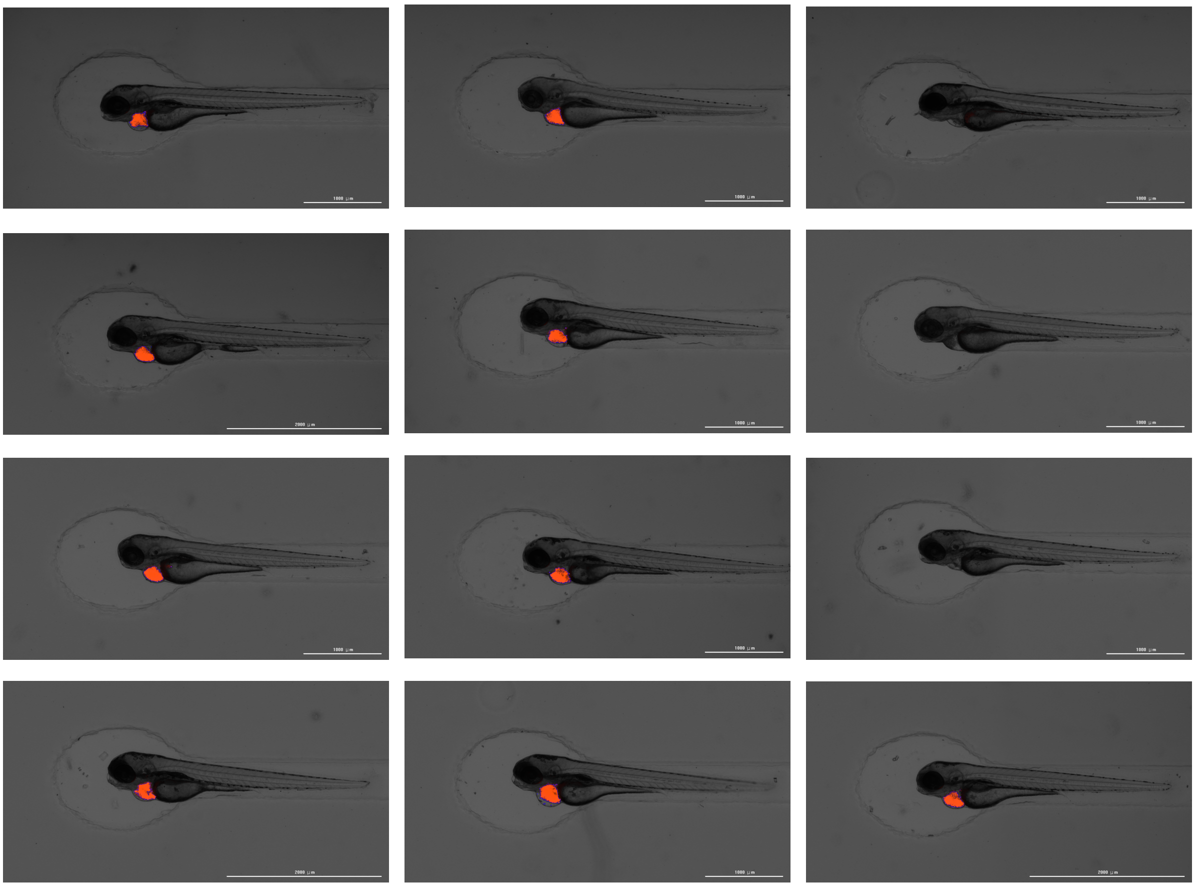

S5 Fig. Representative fluorescence images of HCT116 injected into the PCS at 1 dpi. Larvae injected with HCT116 into the PCS using the automated microinjection system were imaged using the TRITC filter of an automated microscope (BioTek Lionheart FX, Agilent). All larvae shown in S5 Fig. were obtained from a single technical replicate, in which successful injection was observed in 9 out of 12 larvae. A video of the injection procedure is available in S4 Movie.
