# Supplementary material for: Automation in microinjection for zebrafish pericardial space with image-based motion control and batch agarose microplate
Source: PLoS One. 2025 Oct 9;20(10):e0333369. doi: 10.1371/journal.pone.0333369 (PMC12510664; doi:10.1371/journal.pone.0333369)
Supplement: S6 Fig — Larvae injected with HCT116 into the PCS using the automated microinjection system were imaged using the TRITC filter of an automated microscope (BioTek Lionheart FX, Agilent). All larvae shown in S6 Fig. were obtained from a single technical replicate, in which all 12 larvae were alive. Engraftment was considered successful only when the fluorescent area at 4 dpi was maintained or increased relative to the area at 1 dpi. In this technical replicate, successful engraftment was observed in 9 out of 12 larvae. https://osf.io/q5v3c/files/osfstorage/68c92197886836d6c61e481b (PDF) [file pone.0333369.s013.pdf]

S6 Fig.

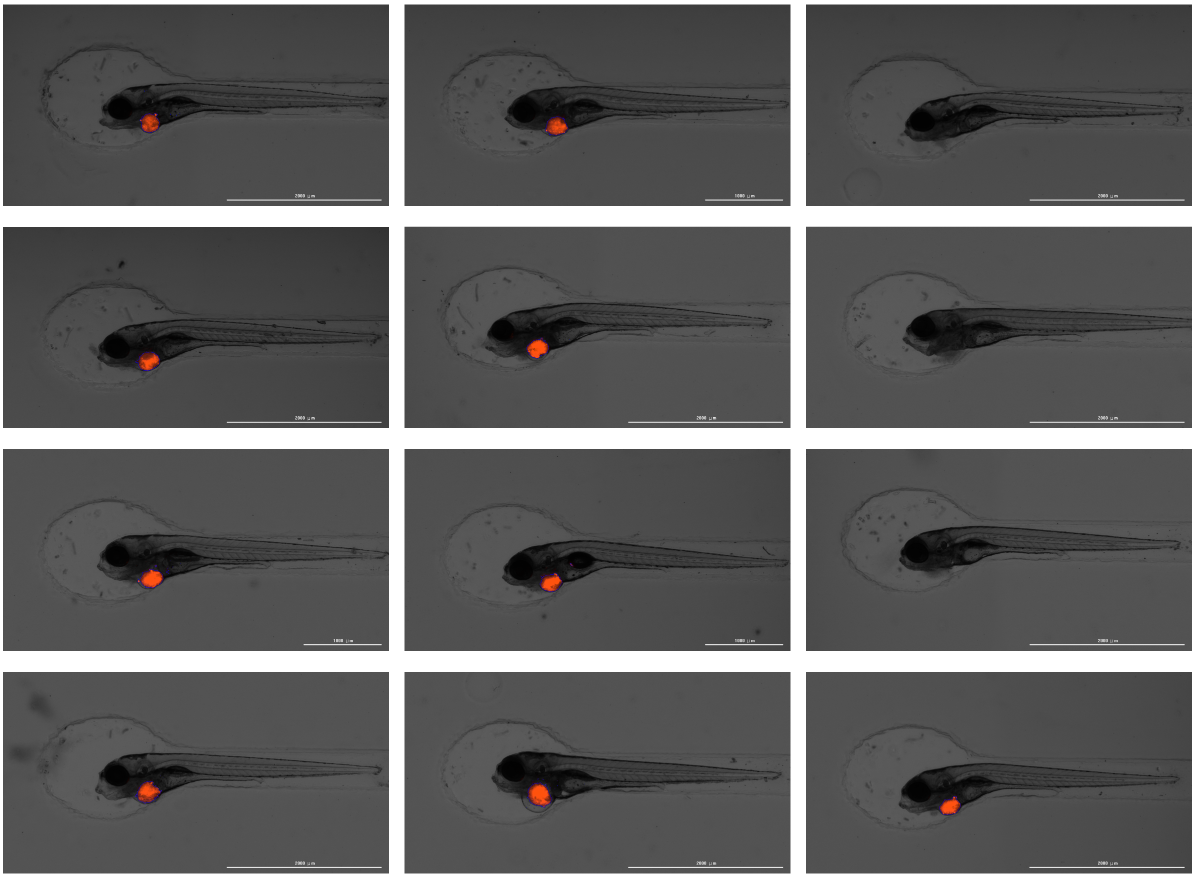

S6 Fig. Representative fluorescence images of HCT116 injected into the PCS at 4 dpi. Larvae injected with HCT116 into the PCS using the automated microinjection system were imaged using the TRITC filter of an automated microscope (BioTek Lionheart FX, Agilent). All larvae shown in S6 Fig. were obtained from a single technical replicate, in which all 12 larvae were alive. Engraftment was considered successful only when the fluorescent area at 4 dpi was maintained or increased relative to the area at 1 dpi. In this technical replicate, successful engraftment was observed in 9 out of 12 larvae.
