# Supplementary material for: Automation in microinjection for zebrafish pericardial space with image-based motion control and batch agarose microplate
Source: PLoS One. 2025 Oct 9;20(10):e0333369. doi: 10.1371/journal.pone.0333369 (PMC12510664; doi:10.1371/journal.pone.0333369)
Supplement: S8 Fig — Larvae injected with SW620 into the PCS using the automated microinjection system were imaged using the TRITC filter of an automated microscope (BioTek Lionheart FX, Agilent). All larvae shown in S8 Fig. were obtained from a single technical replicate, in which 11 out of 12 larvae were alive. Engraftment was considered successful only when the fluorescent area at 4 dpi was maintained or increased relative to the area at 1 dpi. In this technical replicate, successful engraftment was observed in 10 out of 11 larvae. https://osf.io/q5v3c/files/osfstorage/68c9219c64ba17344e53d8aa (PDF) [file pone.0333369.s015.pdf]

**S8 Fig.**

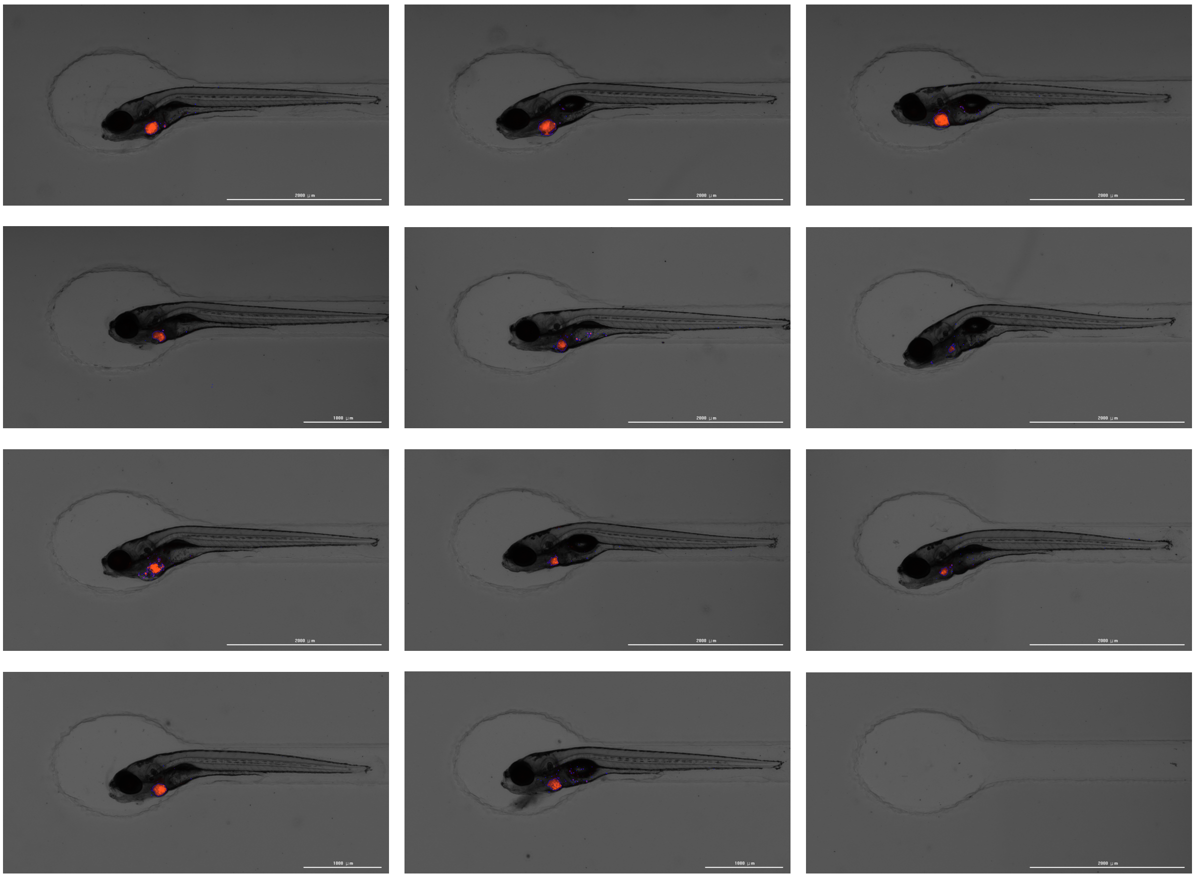

**S8 Fig. Representative fluorescence images of SW620 injected into the PCS at 4 dpi.** Larvae injected with SW620 into the PCS using the automated microinjection system were imaged using the TRITC filter of an automated microscope (BioTek Lionheart FX, Agilent). All larvae shown in S8 Fig. were obtained from a single technical replicate, in which 11 out of 12 larvae were alive. Engraftment was considered successful only when the fluorescent area at 4 dpi was maintained or increased relative to the area at 1 dpi. In this technical replicate, successful engraftment was observed in 10 out of 11 larvae.
